# Cell division cycle‐associated 8 is a prognostic biomarker related to immune invasion in hepatocellular carcinoma

**DOI:** 10.1002/cam4.5718

**Published:** 2023-02-28

**Authors:** Haomin Wu, Shiqi Liu, Di Wu, Haonan Zhou, Guoxin Sui, Gang Wu

**Affiliations:** ^1^ Department of General Surgery The First Hospital of China Medical University Shenyang China

**Keywords:** biomarker, CDCA8, HCC, immune infiltration, prognosis

## Abstract

**Background:**

Cell division cycle‐associated 8 (CDCA8) is involved in numerous signaling networks, and it serves a crucial modulatory function in multiple malignant tumors. However, its significance in prognosis and immune infiltration in hepatocellular carcinoma (HCC) remains unclear.

**Materials and methods:**

Herein, we examined the CDCA8 levels in tumor tissues, as well as its associated signaling pathways and correlation with immune infiltration. Additionally, we further clarified the prognostic significance of CDCA8 among HCC patients. HCC patient information was recruited from The Cancer Genome Atlas (TCGA). Using bioinformatics, the following parameters were analyzed among HCC patients: CDCA8 expression, enrichment analysis, immune infiltration, and prognosis analysis. Moreover, we employed in vitro investigations, such as, qRT‐PCR, immunohistochemistry (IHC), and cell functional experiments to validate our results.

**Results:**

Elevated CDCA8 expression in HCC patients was markedly associated with T stage, pathological status (PS), tumor status (TS), histologic grade (HG), and AFP. Elevated CDCA8 expression HCC patients exhibited reduced overall survival (OS) (*p* < 0.001), disease‐specific survival (DSS) (*p* < 0.001), and progress free interval (PFI) H(*p* < 0.001). According to the ROC analysis, the area under the curve (AUC) was 0.997. Multivariate analysis revealed that CDCA8 was a stand‐alone prognostic indicator of patient OS (*p* = 0.009) and DSS (*p* = 0.006). A nomogram was then generated based on the multivariate analysis, and the C‐indexes and calibration chart revealed excellent predictive performance in determining HCC patient outcome. Based on the GSEA analysis, CDCA8 modulated the P53, Notch, PPAR, E2F networks. We observed a direct link between CDCA8 levels and Th2 and T helper cells, and a negative link between CDCA8 levels and dendritic cells (DC), neutrophils, cytotoxic cells, and CD8 T cells. Furthermore, CDCA8 deficiency inhibited liver cancer cell proliferation and invasion.

**Conclusion:**

In conclusion, these findings indicate that CDCA8 is a new molecular bioindicator of HCC patient prognosis, and it is an excellent candidate for therapeutic target against HCC.

## Graphical abstract

Functions of CDCA8 in hepatocellular carcinoma. The expression of CDCA8 regulated the expression of tumor related proteins such as P53, PPAR, MYC etc. High level of CDCA8 activating the Notch pathway. Moreover, CDCA8 take part in the cell cycle and related to immune invasion. High level of CDCA8 means bad tumor status and poor survival in patients.

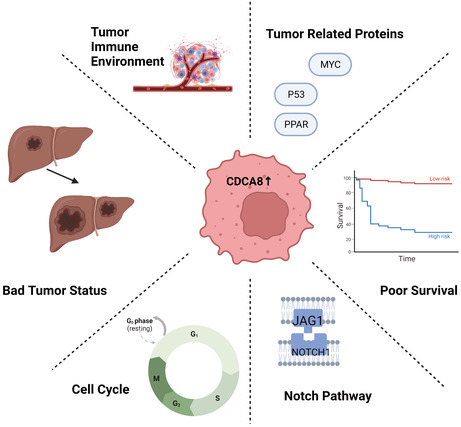

## 1 INTRODUCTION

Hepatocellular carcinoma (HCC) is a widespread malignancy with the sixth and fourth leading cases of morbidity and mortality worldwide.^1^ Thus far, HCC treatment has seen massive advances; however, the postoperative tumor recurrence and metastasis rate remains relatively high and prognosis quite poor.^2^, ^3^ HCC development and progression involves numerous factors and mechanisms.^4^ Additionally, HCC is the third leading contributor to cancer‐associated mortality, which occurs due to the absence of an appropriate early‐detection bioindicator, limited knowledge of the associated molecular mechanisms, and resistance to available chemotherapy.^5^ It is, thus, critical to identify new prognostic bioindicators for the early detection and therapeutic targeting of molecules involved in HCC etiology.

An important sign of tumor development is the disruption of normal cell cycle regulation.^6^, ^7^ In fact, all CDCAs, particularly, CDCA2, CDCA3, CDCA5, and CDCA8, exhibit direct associations with cell cycle and proliferation among almost all cancers.^8^ The same is observed with mitotic cell cycle‐associated genes like Cdc123, CDCA8, Ccnb2, Psmd8, Pola1, Nde1, Anapc1, and Anapc5.^9^ CDCA8 is a potential oncogene, which is markedly elevated in numerous cancers, and it is critical for the survival and malignancy of different cancer cells,^10^ as well as indicate poor prognosis and participate in tumorigenesis, particularly, in the malignant glioma,^11^ ovarian cancer,^12^ lung adenocarcinoma,^13^ and so on. The CDCA8 protein forms a major portion of the vertebrate chromosomal passenger complex (CPC), which consists of four other proteins, namely, borealin (CDCA8), survivin (BIRC5), inner centromere protein (INCENP), and aurora kinase B (AURKB).^14^ Moreover, the complex localizes to the mitotic chromosomes during metaphase, where it modulates chromosome condensation and the spindle assembly checkpoint.^15^ CPC component protein overexpression strongly impairs CPC activity, along with mitotic repair and modulation. This, in turn, results in aberrant cell division and aneuploidy, which accelerates the origination and development of malignant tumors.^16^, ^17^

The tumor immune microenvironment (TIME) is a newly reported concept, which is intricately linked to tumor patient prognosis.^18^ Distinctive immune cell populations, namely, innate and adaptive immune cells like Th2, T helper, CD8 T, neutrophils, cytotoxic, and DC are typically present in the TIME.^19^ Infiltrated immune cells in the tumor microenvironment (TME) serve an essential function in tumorigenesis and tumor progression.^20^ Prior research revealed that HCC primarily occurs after liver damage and widespread inflammation, hence, it occurs alongside immune cell invasion.^21^ Despite multiple investigation identifying high levels of CDCA8 in HCC, as well as its association with tumor infiltration and metastasis,^22^, ^23^ the correlation between CDCA8 levels and immune infiltration remains unclear. However, given its large accumulation in HCC, it is worth extra attention and exploration as a novel and significant prognostic biomarker of HCC.

Herein, our goal was to extensively evaluate CDCA8 significance in HCC using the TCGA database‐based RNA sequencing (RNA‐seq) data and bioinformatics and statistical analyses. Our utilization of differentially expressed genes (DEG), as well as gene ontology (GO), Kyoto Encyclopedia of Genes and Genomes (KEGG) network, gene set enrichment (GSEA), single‐sample gene set enrichment (ssGSEA), Kaplan–Meier (KM) survival, and logistic & Cox regression analyses provided us with an overall understanding of the CDCA8 role in HCC. Based on our investigation, CDCA8 was strongly upregulated in HCC, and it accelerated tumor cell proliferation and infiltration. Moreover, CDCA8 was closely related to HCC patient prognosis, and was a stand‐alone prognostic indicator. Meanwhile, CDCA8 enrichment analysis revealed that CDCA8 modulated the NOTCH, PPAR, P53, MYC‐ACTIVE, E2F, ATM and other signaling networks. Hence, we demonstrated that CDCA8 was strongly associated with immune infiltration.

## 2 MATERIALS AND METHODS

### 2.1 Date source and processing

**FIGURE 1:**
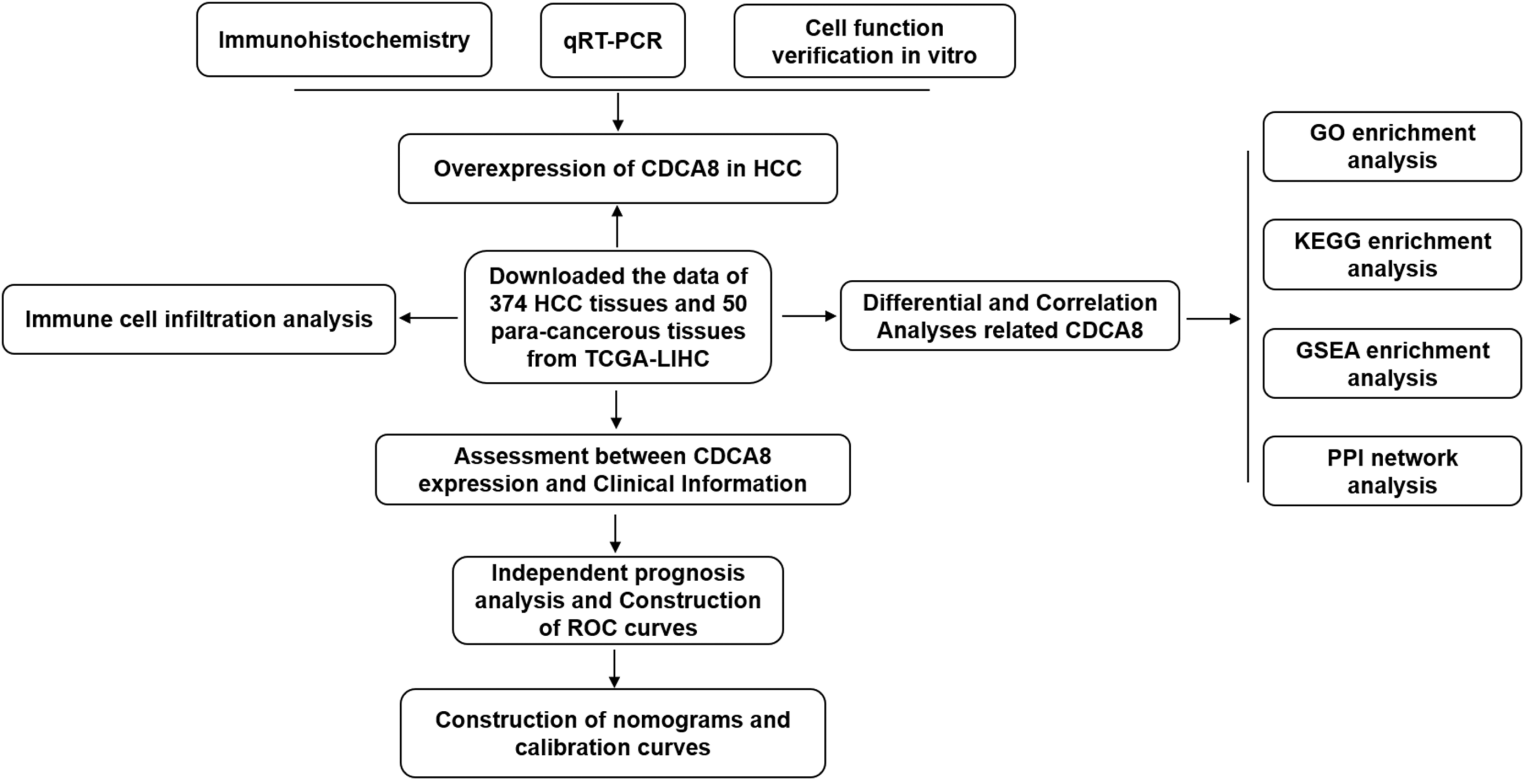
Schematic representation of the workflow with details of step sequences.

The detailed workflow of our study is shown in (Figure 1). The Cancer Genome Atlas (TCGA), a well‐established cancer genomic program, identifies more than 20,000 primary cancer and corresponding normal samples across 33 cancer categories (https://cancergenome.nih. gov/). CDCA8 expression data with corresponding clinical information (50 para‐cancerous tissue and 374 HCC tissues) were retrieved from the TCGA database. Clinical characteristic data, namely, T and M stages, pathologic stage (PS), tumor status (TS), AFP, histologic grade (HG), age, gender, and vascular invasion were extracted from the TCGA‐LIHC. Data processing was performed, which converted the RNA‐seq data in FPKM (Fragments Per Kilobase per Million) to TPM (transcripts per million reads) format, prior to log2 conversion. Eventually, the R software (version 3.6.3) and R package (ggplot2) were employed for visual analysis. The Human Protein Atlas (HPA) offers 26,000 human protein data across various tissues and cellular distributions. Individual protein profiles in 64 cell lines, as well as 48 normal human and 20 tumor tissues were assessed via immunoassay and the database. To further clarify CDCA8 protein expression, we retrieved relevant information from HPA (https://www.proteinatlas.org/).

### 2.2 Differential and correlation analyses

Differentially expressed genes (DEGs) between reduced and elevated CDCA8 expressions (reduced:0%–50%, elevated:50%–100%) patients from the TCGA datasets were screened using the unpaired Student's t‐test within the DESeq2 (1.26.0) package.^24^ Genes with an adjusted *p*‐value <0.01 and absolute FC >2 were set as the significance threshold. All DEGs are expressed in volcano plots. LinkedOmics (http://www.linkedomics.org/) is an open access website that contains multi‐omics information from all 32 TCGA cancer types and 10 clinical proteomics tumor analysis consortium (CPTAC) cancer populations. This one‐of‐a‐kind platform facilitates the availability, analysis, and comparison of cancer multi‐omics information within and across tumor types. We applied this public database for correlation analysis. The selected cancer cohort was TCGA‐LIHC, and the data type was RNA‐seq. All positively and negatively correlated genes with significant differences are presented on the heatmap.

### 2.3 Functional Enrichment (FEA) and immune cell invasion assessment

Metascape (http://metasape.org) was employed for CDCA8‐associated DEG enrichment analysis,^25^ with parameters as follows: *p* < 0.01, at least a count of 3, and enrichment factor >1.5 to achieve marked statistical differences. GSEA was conducted via the GSEA version 3.0 and R package clusterProfiler.^26^ The gene set “c2.cp.v7.2.symbols.gmt,” which was employed as a reference gene set, was retrieved from the Molecular Signatures Database (MSigDB) (http://software.broadinstitute.org/gsea/msigdb). A singular gene set was deemed markedly enriched when the *p*‐value was <0.05 and FDR was <0.25. The relative tumor invasion profile of 24 immune cell types were assessed via the ssGSEA algorithm and R package GSVA.^27^ The Wilcoxon rank sum test and Spearman correlation were employed to assess associations between CDCA8 levels and the immune cell infiltration status.

### 2.4 Clinical statistical analysis

The R software (V3.6.3) was utilized for all data analyses. The CDCA8 levels were stratified as either elevated or reduced based on the median, and the correlation between patient clinical profile and CDCA8 levels was evaluated via the following statistical approaches, namely the chi‐squared, Fisher's exact, and the Wilcoxon rank sum tests. We also performed logistic regression analysis. The receiver operating characteristic (ROC) curves was employed for the diagnostic performance assessment of CDCA8. Potential correlations between the clinicopathological profile and the overall survival (OS), progression free interval (PFI), and disease‐specific survival (DSS) in the TCGA were examined via the KM analysis. Uni‐ and Multivariate Cox analyses, in combination with patient clinical profile, were employed for the comparison of CDCA8 impact on OS. Statistically significant prognostic indicators from the univariate analysis were entered into the multivariate analysis, and the corresponding relevant risk factors were used to generate 1, 3, and 5‐year patient prognosis estimation nomograms. The nomograms were generated with the survival package, and they included marked clinical profiles and calibration plots. Graphs were used to assess the calibration curves via mapping the nomogram‐estimated probabilities against the actual observations, and the 45°line represented the optimal estimation.

### 2.5 Tissues and cell culture

Eighty HCC tissues and corresponding noncancerous adjoining tissues (NATs) were harvested from HCC patients who received surgical intervention at the First Affiliated Hospital of China Medical University (Shenyang, China) between 2018 and 2019. All participants were pathologically confirmed post‐surgery, and signed an informed consent form. NATs were extracted from regions over 2 cm away from the tumor. All tissue samples were quickly flash frozen in liquid nitrogen following harvest, and before transfer to −80°C for prolonged storage. This work received ethical approval from our institution. All liver cancer cells, namely, Bel7402, Huh7, HCCLM3, SMMC7721, HepG2, and LO2, were acquired from the Shanghai Cell Bank (Shanghai, China). All cells were grown in high‐glucose Dulbecco's‐modified eagle medium (HyClone) with 10% fetal bovine serum (FBS) and 1% penicillin/streptomycin at 37°C in a 5% CO2 incubator (Thermo).

### 2.6 RNA isolation and quantitative real‐time PCR (qRT‐PCR)

Total RNA was isolated from HCC tissues and cells using TRIzol reagent (Invitrogen), as per kit directions, then converted to complementary DNA (cDNA) via the Mir‐X™ miRNA First‐Strand Synthesis Kit (Clontech) or PrimeScript RT reagent Kit (Takara). TB Green^®^ (RR820A, Takara) was employed for CDCA8 transcript level identification via the Light Cycler 480 II Real‐Time PCR system (Roche Diagnostics). GAPDH served as the internal control: Forward: CCGTGAAGTGGAAATACGAATC. Reverse: GGATCTCGATGTTGTAGAGGTT. All samples were examined thrice, and the melting curve analysis was employed for primer specificity evaluation. Cycle threshold (Ct) was determined according to the total number of cycles required for the TB Green^®^ signal to cross the threshold. The relative CDCA8 levels were computed via Ct. The employed primers were designed and synthesized by Sangon Biotech. The fold‐change in transcript levels was computed via the 2 − ΔΔCt formula. The CDCA8 primers were as follows: Forward: CCGTGAAGTGGAAATACGAATC. Reverse: GGATCTCGATGTTGTAGAGGTT.

### 2.7 Immunohistochemistry (IHC)

CDCA8 expressions in 20 HCC patients were assessed using paraffin‐embedded specimens, which were cut into four‐μm thick sections, followed by deparaffinization in xylene, and dehydration for 5 min in an autoclave, prior to antigen retrieval. Hydrogen peroxide (0.3%) was employed to block the cellular peroxidase activity, and normal goat serum for 30 min at 37 °C was used to block non‐specific immunoglobulin interaction locations. Subsequently, the specimens were overnight (ON) treated with anti‐CDCA8 at 4°C, followed by treatment with biotinylated goat anti‐rabbit IgG secondary antibody (Maixin Kit) for 1 h at room temperature (RT), and lastly, streptavidin–biotin horseradish peroxidase‐conjugated (Maixin Kit) for 30 min at RT. The peroxidase reaction was developed using 3′‐diaminobenzidine tetrahydrochloride (Maixin Kit).

### 2.8 Cell transfection

Bel7402 and Huh7 cells, with relatively high CDCA8 expressions, were employed for viral incorporations. CDCA8‐shRNA lentiviral transfection reagent was purchased from GENE. We employed three distinct target transfection reagents, and one negative control (NC). The target sequence of CDCA8‐sh1, CDCA8‐sh2, CDCA8‐sh3 were cgGAGAGAGCCTGCGATTATT, gtTTGACTCAAGGGTCTTCAA, and ccTGGATATCACCGAAATAAA, respectively. The NC insert sequence was TTCTCCGAACGTGTCACGT. Based on our preliminary results, we selected an MOI of 50 for cellular transfection. MOI represents the ratio of the phages number to viruses during infection. In other words, the average number of phages per infected bacterium. Cell transfection was carried out following the instruction manual of lentiviral transfection. Puromycin (200 nm) was used to further screen for transfected cells.

### 2.9 Cell functional evaluations

5 × 104 CDCA8‐shRNA‐containing cells were plated with 200ul high‐glucose DMEM medium (without FBS) in the top chamber, whereas, 600ul culture medium with 10% FBS only was introduced to the bottom chamber. For the invasion assay, 50 μL Matrigel (1:9, BD Bioscience) was introduced in the transwell chamber (Costar, USA). The chamber was gently rinsed a few times, and the cells inside the chamber were wiped off with a cotton swab after a 48 h incubation at 37°C with 5% CO2. Subsequently, cells on the basement membrane of the chamber received hematoxylin and eosin staining prior to imaging under a Leica DM3000 microscope (Leica). Stained cell quantification was done via the color grab comparison tool of the Image‐Pro6.0 software.

### 2.10 Statistical analysis

The SPSS 21.0 software was employed for all data analyses, and the data from ≥3 distinct experiments are provided as mean ± standard deviation (SD). Inter‐group differences were examined via Student's *t*‐test. *p* < 0.05 was set as the significance threshold.

## 3 RESULTS

### 3.1 CDCA8 is elevated in HCC tissues

**FIGURE 2:**
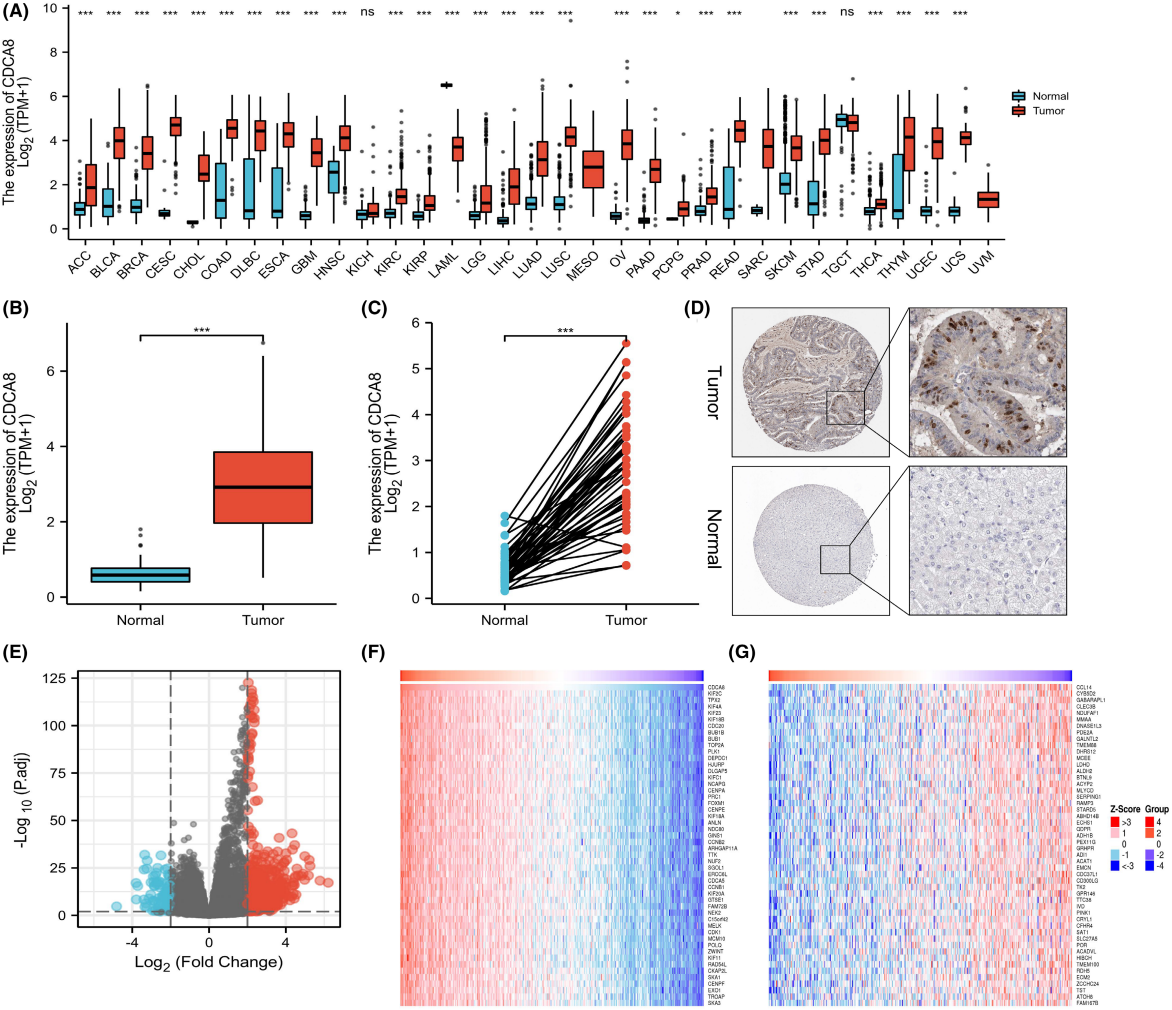
Expression of Cell division cycle associated 8 (CDCA8) in different malignant tumors and analysis of its differences and related genes from The Cancer Genome Atlas. (A) Expression of CDCA8 in unpaired pan‐carcinoma. (B) Expression of CDCA8 in unpaired hepatocellular carcinoma (HCC) samples. (C) Expression of CDCA8 in paired HCC samples. (D) The protein level expression of CDCA8 from Human Protein Atlas (HPA). (E) Analysis of CDCA8 differences genes. (F) Genes that were positively correlated with CDCA8. (G) Genes that were negatively correlated with CDCA8.

**FIGURE 3:**
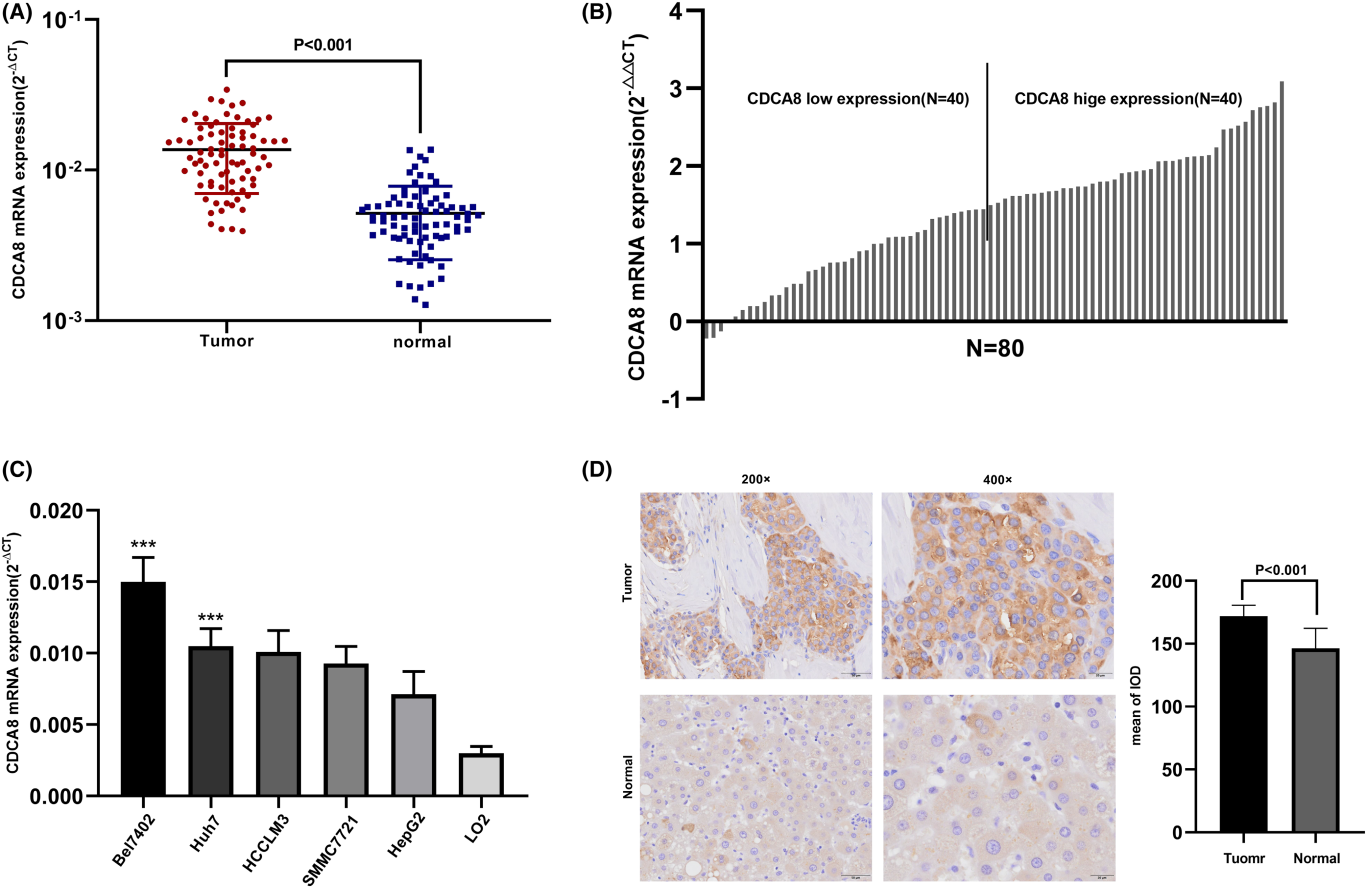
Expression of Cell division cycle associated 8 (CDCA8) in hepatocellular carcinoma (HCC) tissues and cell lines. (A) Expression level (2^−△△CT^) of CDCA8 was quantified in HCC tissues compared with adjacent tissues among 80 HCC patients by qRT‐PCR. (B) Expression level (2^−△CT^) of CDCA8 was quantified in HCC tissues compared with adjacent tissues among 80 HCC patients by qRT‐PCR. (C) Expression of CDCA8 in cell lines. ***p < 0.01. (D) Immunohistochemical evaluation of CDCA8 protein expression.

Firstly, we assessed CDCA8 expressions in 15,776 unpaired tumor and normal samples in pan‐cancer. CDCA8 was highly expressed in various malignant tumors, including adrenocortical carcinoma (ACC), cholangiocarcinoma (CHOL), renal clear cell carcinoma (KIRC), and so on (*p* < 0.05) (Figure 2A). Additionally, comparable results were obtained from the paired tissue samples (Figure S1). Next, we evaluated CDCA8 levels in 50 para‐cancerous and 374 HCC samples, paired and unpaired using the TCGA‐LIHC dataset. Based on our analysis, the CDCA8 levels were markedly elevated in HCC samples (*p* < 0.001) (Figure 2B,C). The CDCA8 protein levels in HCC and normal tissues, obtained from the HPA, are presented in Figure 2D. Subsequently, we conducted CDCA8 DEG analysis, based on the CDCA8 median expression grouping, which revealed 776 highly expressed and 99 scarcely expressed genes (|log2(FC)| > 2 & *p* adj <0.05) (Figure 2E). We then applied the linkedOmics online database to assess the positive and negative correlations of CDCA8 genes (Figure 2F). Lastly, we further validated the markedly elevated CDCA8 expression in HCC tissues using qt‐PCR, western‐blot, and IHC (Figure 3A,B,D,E). Among all HCC cells, CDCA8 transcript levels were substantially elevated in the BEL‐7402 and HUH‐7 cells. Thus, these cells were selected for subsequent experiments (Figure 3C).

### 3.2 CDCA8 associated with multiple signal pathways and cell functions

**FIGURE 4:**
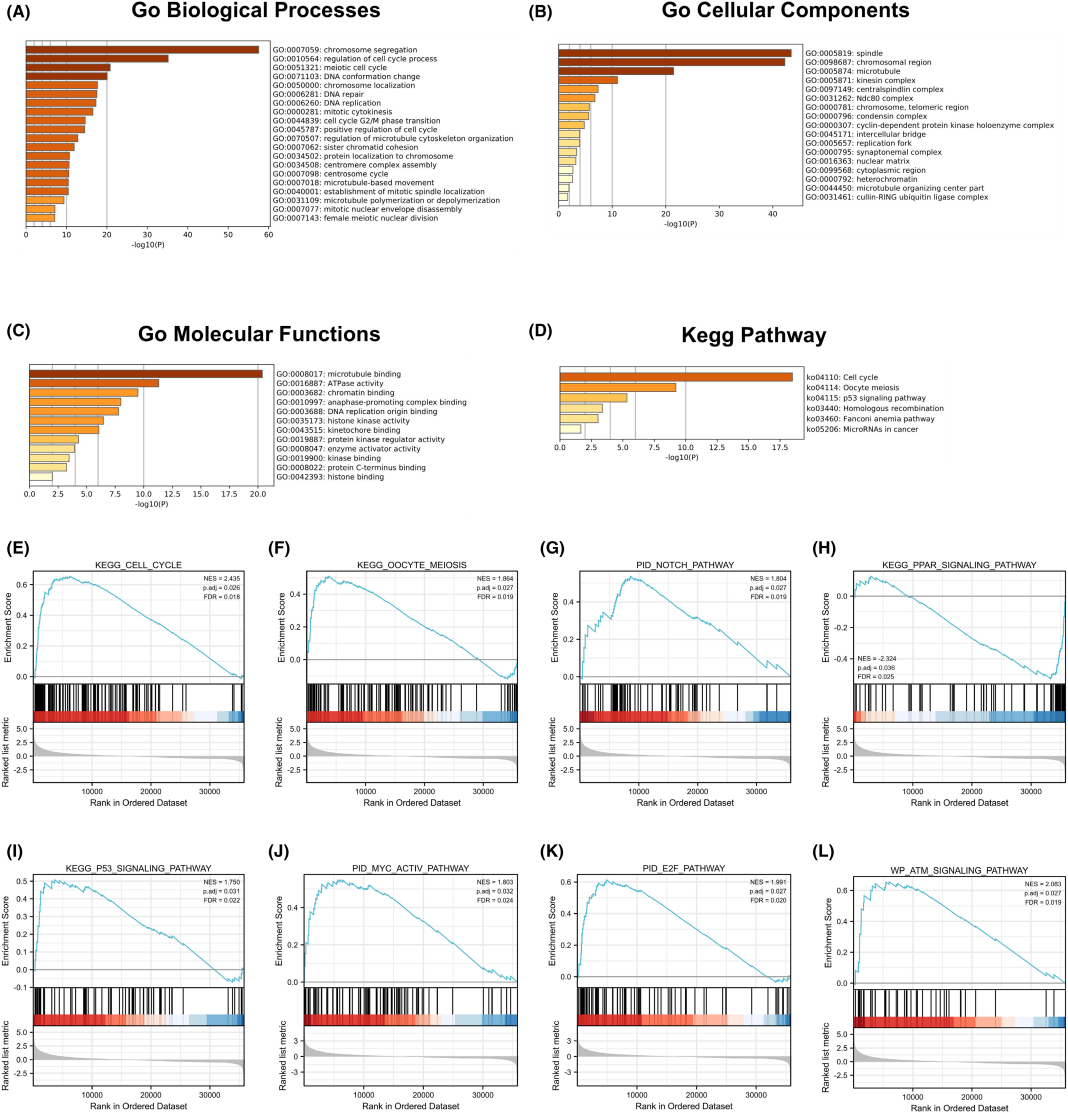
Gene enrichment analysis for Cell division cycle associated 8 (CDCA8). (A) gene ontology (GO) enrichment analysis of CDCA8 in biological processes. (B) GO enrichment analysis of CDCA8 in cellular components. (C) GO enrichment analysis of CDCA8 in molecular functions. (D) Six signaling pathways are enriched from Kyoto Encyclopedia of Genes and Genomes pathway about CDCA8. (E–L) Enrichment plots from the gene set enrichment analysis (GSEA). Several pathways and biological processes were differentially enriched in CDCA8‐related HCC. (E) Cell cycle. (F) Oocyte meiosis. (G) Notch pathway. (H) PAR.I:P53 signaling pathway. (J) MYC‐active pathway. (K) E2F pathway. (L) ATM signaling pathway.

To elucidate the functional pathways related to the 451 CDCA8 DEGs, we employed Metascape for GO and KEGG enrichment analyses. Our analysis revealed that CDCA8‐associated genes modulated multiple biological processes (BPs), cellular compositions (CCs), molecular functions (MFs), and KEGG networks (Figure 4A‐D). Using the MSigDB enrichment analysis, we revealed marked differences between the elevated and reduced CDCA8 level datasets. The major enrichment pathways included cell cycle, meiosis, NOTCH, PPAR, P53, MYC‐ACTIVE, E2F, and ATM axes (Figure 4E–L). To discern potential associations between the 451 DEGs in HCC patients, a PPI network was generated using Metascape. We identified 10 protein interaction networks associated with this gene (Figure S2).

### 3.3 CDCA8 expression correlated with immune invasion

**FIGURE 5:**
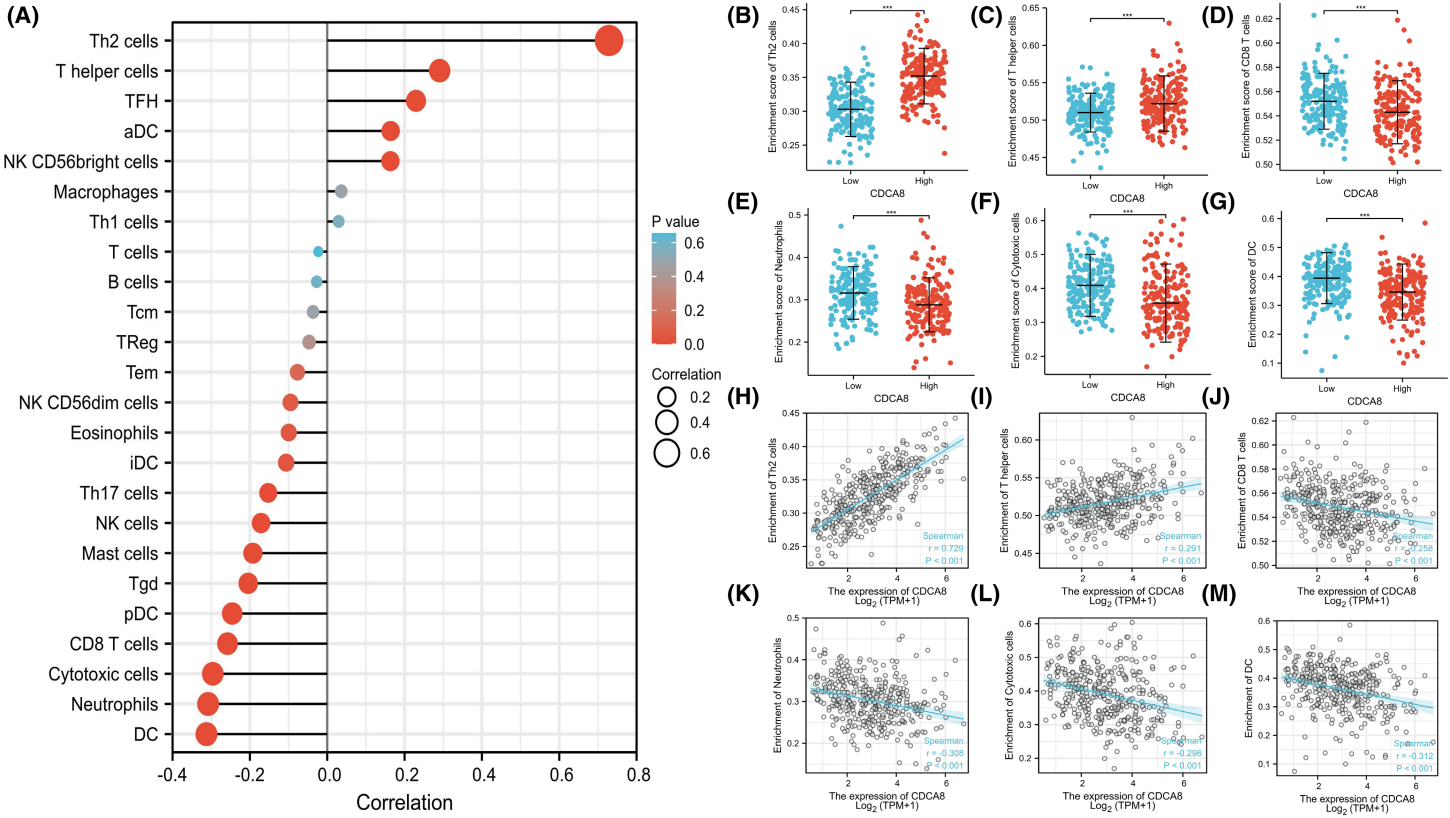
Expression level of Cell division cycle associated 8 (CDCA8) in hepatocellular carcinoma (HCC) was related to immune infiltration in tumor microenvironment. (A) The correlation between CDCA8 and 24 kinds of immune cells was demonstrated by using the bar graph, in which CDCA8 and Th2, T helper, CD8 T, Cytotoxic cells, Neutrophils and DC were significantly correlated. (B) Scatter plots showed the difference of Th2 cells level between CDCA8‐high/−low groups. (C) Scatter plots showed the difference of T helper cells level between CDCA8‐high/−low groups. (D) Scatter plots showed the difference of CD8 T cells level between CDCA8‐high/−low groups. (E) Scatter plots showed the difference of Neutrophils level between CDCA8‐high/−low groups. (F) Scatter plots showed the difference of Cytotoxic cells level between CDCA8‐high/−low groups. (G) Scatter plots showed the difference of DC infiltration level between CDCA8‐high/−low groups. (H) Correlation analysis between Th2 cells and CDCA8. (I) Correlation analysis between T helper cells and CDCA8.(J) Correlation analysis between CD8 T cells and CDCA8. (K) Correlation analysis between Neutrophils and CDCA8. (L) Correlation analysis between Cytotoxic cells and CDCA8. (M) Correlation analysis between DC infiltration and CDCA8.

The link between CDCA8 expression and ssGSEA‐quantified immune cell invasion level was assessed via Spearman correlation. Based on our analysis, CDCA8 expression was positively correlated with Th2 and T helper cells, and negatively correlated with CD8 T, neutrophils, DC, and cytotoxic cells (*p* < 0.001) (Figure 5A‐M).

### 3.4 CDCA8 expression correlated with clinical information

**FIGURE 6:**
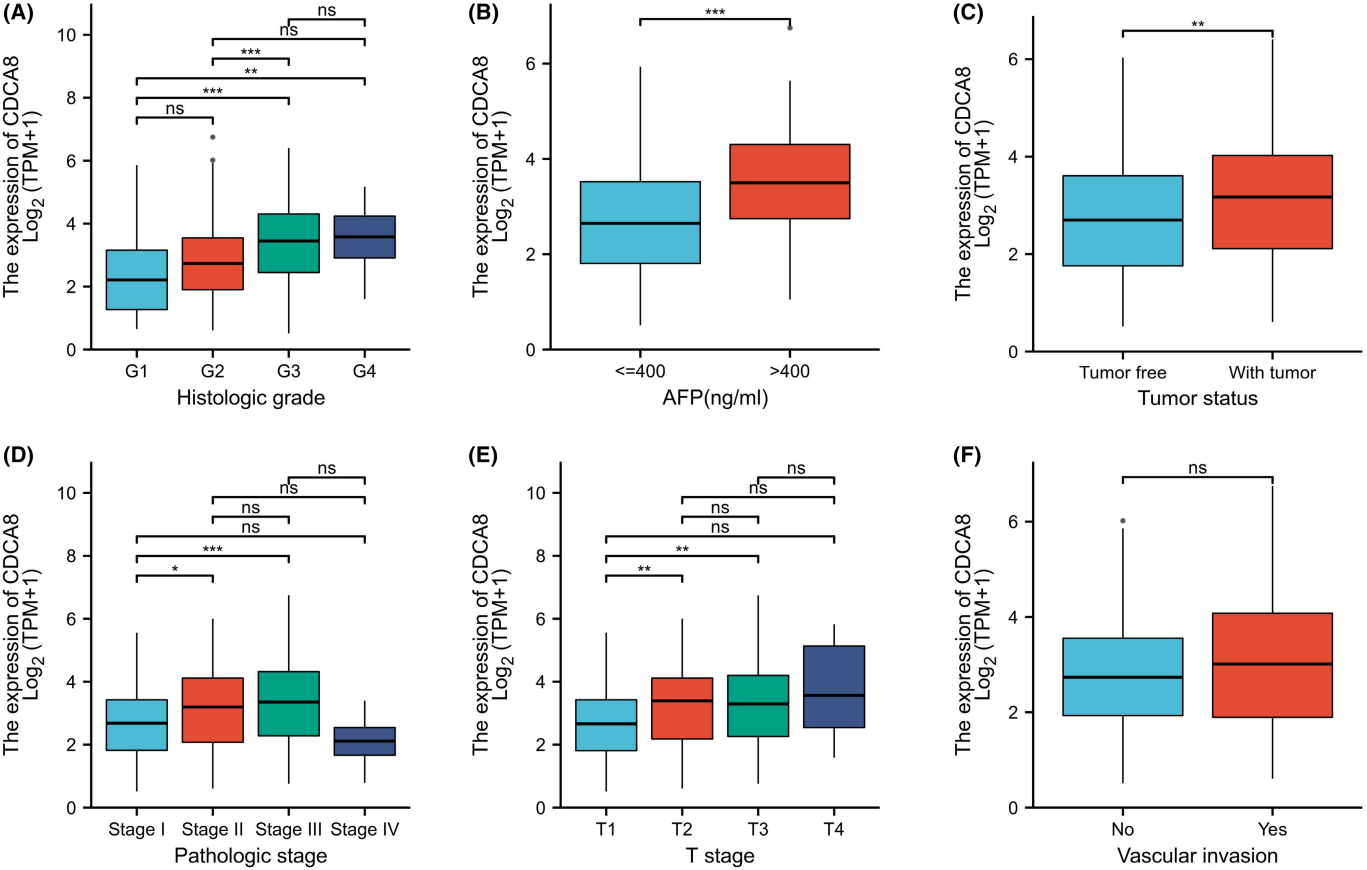
Relationship between Cell division cycle associated 8 (CDCA8) expression and clinicopathological characteristics(date source:TCGA). (A) T stage. (B) Pathologic stage. (C) Tumor status. (D) Histologic grade. (E) AFP. (F) Vascular invasion.

To elucidate the putative CDCA8 function in HCC patients, we analyzed 424 HCC samples with CDCA8 expression profile and corresponding patient clinical profile from TCGA. As depicted in Table 1, CDCA8 expression was strongly associated with T stage (*p* = 0.002), PS (*p* = 0.002), TS (*p* = 0.007), HG (*p* < 0.001), and AFP (*p* < 0.001) (Figures 6A–E). However, CDCA8 levels showed no association with vascular invasion (Figures 6F). Using univariate analysis, we demonstrated that CDCA8 levels were intricately linked to worse prognostic clinicopathological profile (Table 2). The results showed that patients with high CDCA8 expression tend to having higher T stage (OR = 1.698 for T3 & T4 vs. T1 & T2), higher PS (OR = 1.750 for Stage III & IV vs. Stage I & II), higher HG (OR = 2.616 for G3 & G4 vs. G1 & G2) and poorer TS (OR = 1.838 for with tumor vs. tumor free). The AFP level is higher in the patients with CDCA8 elevated(OR = 3.247 for >400 ng/mL vs. <=400 ng/mL).

**TABLE 1:** The association between CDCA8 expression and clinicopathological variables.

Characteristic	Low expression of CDCA8	High expression of CDCA8	*p*‐Value
*n*	187	187	
T stage, *n* (%)			**0.002** **
T1	109 (29.4%)	74 (19.9%)	
T2	38 (10.2%)	57 (15.4%)	
T3	32 (8.6%)	48 (12.9%)	
T4	5 (1.3%)	8 (2.2%)	
N stage, *n* (%)			0.622
N0	125 (48.4%)	129 (50%)	
N1	1 (0.4%)	3 (1.2%)	
M stage, *n* (%)			0.369
M0	132 (48.5%)	136 (50%)	
M1	3 (1.1%)	1 (0.4%)	
Pathologic stage, *n* (%)			**0.002** **
Stage I	102 (29.1%)	71 (20.3%)	
Stage II	38 (10.9%)	49 (14%)	
Stage III	32 (9.1%)	53 (15.1%)	
Stage IV	4 (1.1%)	1 (0.3%)	
Tumor status, *n* (%)			**0.007** **
Tumor free	115 (32.4%)	87 (24.5%)	
With tumor	64 (18%)	89 (25.1%)	
Gender, *n* (%)			0.269
Female	55 (14.7%)	66 (17.6%)	
Male	132 (35.3%)	121 (32.4%)	
Age, *n* (%)			0.380
<=60	84 (22.5%)	93 (24.9%)	
>60	103 (27.6%)	93 (24.9%)	
Residual tumor, *n* (%)			0.460
R0	169 (49%)	158 (45.8%)	
R1	7 (2%)	10 (2.9%)	
R2	1 (0.3%)	0 (0%)	
Histologic grade, *n* (%)			**<0.001** ***
G1	38 (10.3%)	17 (4.6%)	
G2	99 (26.8%)	79 (21.4%)	
G3	45 (12.2%)	79 (21.4%)	
G4	3 (0.8%)	9 (2.4%)	
Adjacent hepatic tissue inflammation, *n* (%)			0.854
None	67 (28.3%)	51 (21.5%)	
Mild	55 (23.2%)	46 (19.4%)	
Severe	11 (4.6%)	7 (3%)	
AFP(ng/ml), *n* (%)			**<0.001** ***
≤400	127 (45.4%)	88 (31.4%)	
>400	20 (7.1%)	45 (16.1%)	
Child‐Pugh grade, *n* (%)			1.000
A	121 (50.2%)	98 (40.7%)	
B	12 (5%)	9 (3.7%)	
C	1 (0.4%)	0 (0%)	
Vascular invasion, *n* (%)			0.210
No	117 (36.8%)	91 (28.6%)	
Yes	53 (16.7%)	57 (17.9%)	
Age, median (IQR)	62 (53, 69)	60.5 (51, 68)	0.296

*Note*: The bold values indicate difference between the two groups has statistical significance.

** *p* < 0.005

*** *p* < 0.001.

**TABLE 2:** CDCA8 expression association with clinical pathological characteristics (logistic regression).

Characteristics	Total (N)	Odds Ratio (OR)	*p*‐Value
T stage (T3 & T4 vs. T1 & T2)	371	1.698 (1.057–2.753)	**0.030** *
N stage (N1 vs. N0)	258	2.907 (0.367–59.196)	0.358
M stage (M1 vs. M0)	272	0.324 (0.016–2.563)	0.331
Pathologic stage (Stage III and Stage IV vs. Stage I and Stage II)	350	1.750 (1.079–2.865)	**0.024** *
Tumor status (With tumor vs. Tumor free)	355	1.838 (1.204–2.820)	**0.005** **
Gender (Male vs. Female)	374	0.764 (0.494–1.179)	0.225
Age (>60 vs. ≤60)	373	0.816 (0.542–1.225)	0.326
Residual tumor (R1 & R2 vs. R0)	345	1.337 (0.515–3.584)	0.551
Histologic grade (G3 & G4 vs. G1 & G2)	369	2.616 (1.695–4.075)	**<0.001** ***
Adjacent hepatic tissue inflammation (Severe vs. Mild and None)	237	0.800 (0.285–2.110)	0.658
AFP(ng/mL) (>400 vs. ≤400)	280	3.247 (1.817–5.975)	**<0.001** ***
Child‐Pugh grade (B&C vs. A)	241	0.855 (0.340–2.065)	0.730
Vascular invasion (Yes vs. No)	318	1.383 (0.870–2.202)	0.171

*Note*: The bold values indicate difference between the two groups has statistical significance.

* *p* < 0.05

** *p* < 0.005

*** *p* < 0.001.

### 3.5 Elevated CDCA8 levels was intricately linked to worse HCC patient prognosis

**FIGURE 7:**
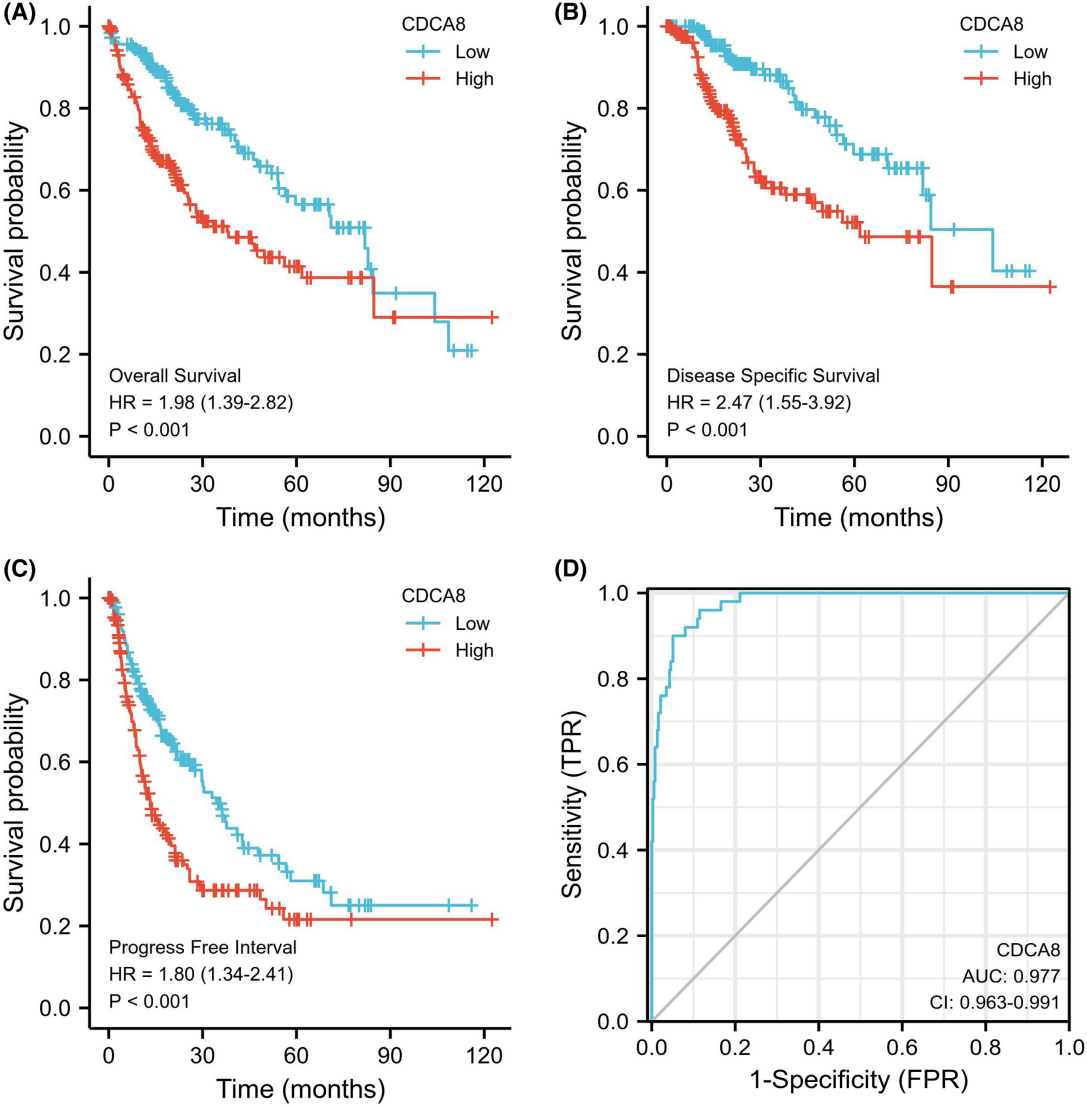
Prognosis and diagnostic efficacy of Cell division cycle associated 8 (CDCA8) in hepatocellular carcinoma. (A) Higher expression of CDCA8 indicates a worse overall survival. (B) Higher expression of CDCA8 indicates a worse disease specific survival (DSS). (C) Higher expression of CDCA8 indicates a worse PFI. (D) A ROC is used to evaluate the diagnostic value of CDCA8 to identity HCC tissues.

**FIGURE 8:**
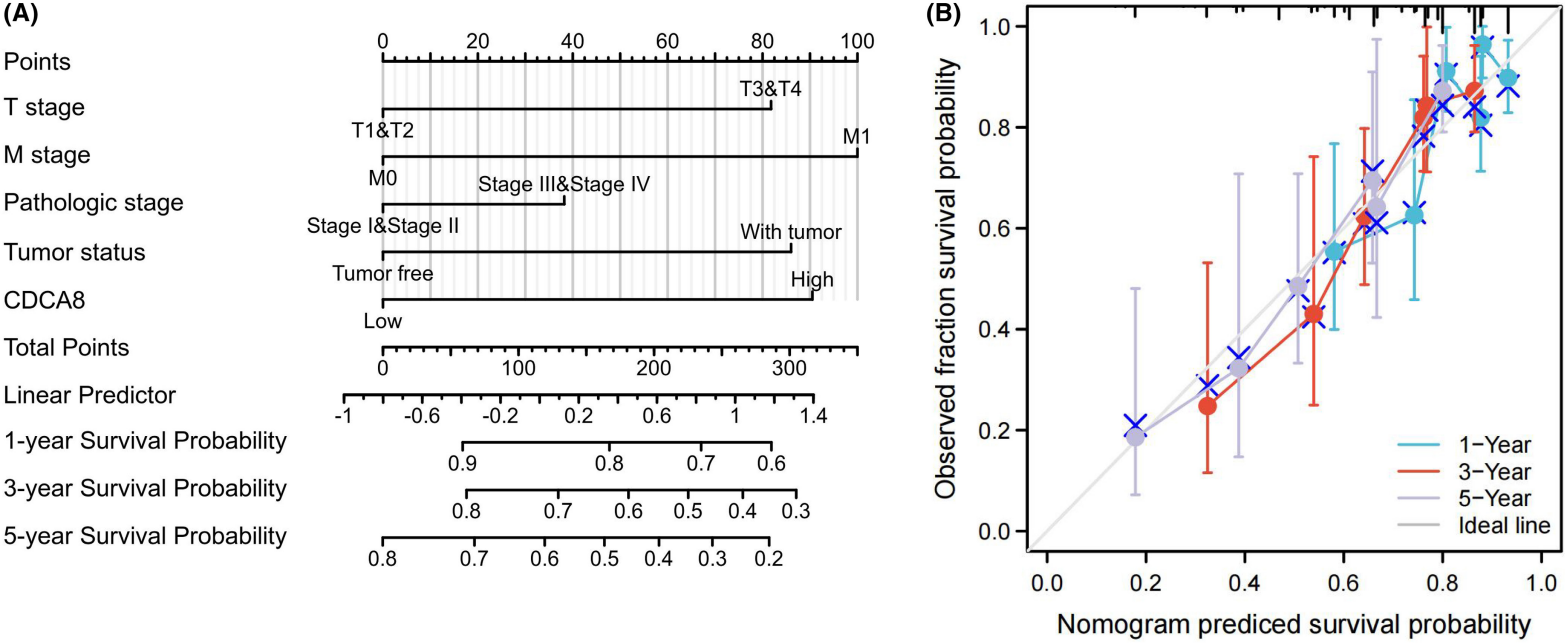
Quantitative method to predict hepatocellular carcinoma (HCC) patients' probability of 1‐, 3‐, and 5‐year OS. (A) A nomogram for predicting the probability of 1‐, 3‐, and 5‐ year OS for HCC patients. (B) Calibration plots of the nomogram for predicting the probability of OS at 1, 3, and 5 years in HCC.

The OS probability was substantially elevated in reduced CDCA8 patients than enhanced CDCA8 patients (*p* < 0.001) (Figure 7A). Likewise, the PFI and DSS probabilities in the reduced CDCA8 patients were markedly elevated, relative to the enhanced CDCA8 patients (*p* < 0.001; *p* < 0.001) (Figure 7B,C). Subsequently, we performed subgroup OS analyses of OS, DSS, and PFI (Figures [Link], [Link]). Based on our data, CDCA8 provided strong guiding prognostic significance in all subgroups. Moreover, we employed the ROC curve to examine the diagnostic efficiency of CDCA8 (AUC = 0.977; CI: 0.963–0.997) (Figure 7D). Our univariate analysis suggested that enhanced CDCA8 expressions produced shorter OS, as well as poorer PFI and DSS. Based on our multivariate analysis, elevated CDCA8 expression was a stand‐alone risk factor with strong associations with poor OS and DSS. However, CDCA8 levels were not a stand‐alone risk factor for poor PFI (Table 3; Tables [Link], [Link]) in HCC patients. To establish a quantitative approach to HCC patient prognosis prediction, CDCA8 levels and its stand‐alone risk factors, namely, T stage, M stage, PS, and TS were employed to generate a nomogram (Figure 8A). The total points of individual variables were readjusted to a range between 1 and 100. The variable points were then accumulated and recorded as the total score. The OS probabilities among HCC patients at 1, 3, and 5‐year were assessed by drawing a vertical line down from the total point axis to the outcome axis. Thus, for an HCC patient with enhanced CDCA8 risk (85 points), the 1‐, 3, and 5‐year OS probabilities were about <60%, 30%, and <20%. We also evaluated the predictability of the nomogram, and found it to be moderately accurate. The bias‐corrected line in the calibration plot was close to the ideal curve (the 45‐degree line), which indicated good agreement between the estimated and actual observations (Figure 8B).

**TABLE 3:** Univariate and multivariate regression (overall survival) of prognosis in patients with HCC.

Characteristics	Total (N)	Univariate analysis		Multivariate analysis	
		Hazard ratio (95% CI)	*p*‐Value	Hazard ratio (95% CI)	*p*‐Value
T stage	370				
T1&T2	277	Reference			
T3&T4	93	2.598 (1.826–3.697)	**<0.001** ***	1.763 (0.239–13.015)	0.578
N stage	258				
N0	254	Reference			
N1	4	2.029 (0.497–8.281)	0.324		
M stage	272				
M0	268	Reference			
M1	4	4.077 (1.281–12.973)	**0.017** *	2.000 (0.455–8.786)	0.359
Pathologic stage	349				
Stage I and Stage II	259	Reference			
Stage III and Stage IV	90	2.504 (1.727–3.631)	**<0.001** ***	1.303 (0.177–9.597)	0.795
Tumor status	354				
Tumor free	202	Reference			
With tumor	152	2.317 (1.590–3.376)	**<0.001** ***	1.815 (1.131–2.913)	**0.013** *
Histologic grade	368				
G1&G2	233	Reference			
G3&G4	135	1.091 (0.761–1.564)	0.636		
AFP(ng/ml)	279				
<=400	215	Reference			
>400	64	1.075 (0.658–1.759)	0.772		
Vascular invasion	317				
No	208	Reference			
Yes	109	1.344 (0.887–2.035)	0.163		
Gender	373				
Female	121	Reference			
Male	252	0.793 (0.557–1.130)	0.200		
Age	373				
<=60	177	Reference			
>60	196	1.205 (0.850–1.708)	0.295		
Residual tumor	344				
R0	326	Reference			
R1 & R2	18	1.604 (0.812–3.169)	0.174		
Child‐Pugh grade	240				
A	218	Reference			
B&C	22	1.643 (0.811–3.330)	0.168		
Adjacent hepatic tissue inflammation	118				
Mild	101	Reference			
Severe	17	1.056 (0.406–2.744)	0.911		
CDCA8	373				
Low	187	Reference			
High	186	1.982 (1.393–2.820)	**<0.001** ***	1.873 (1.167–3.006)	**0.009** **

*Note*: The bold values indicate difference between the two groups has statistical significance.

* *p* < 0.05

** *p* < 0.005

*** *p* < 0.001.

### 3.6 CDCA8 silencing suppresses liver cancer cell proliferation, invasion, and migration

**FIGURE 9:**
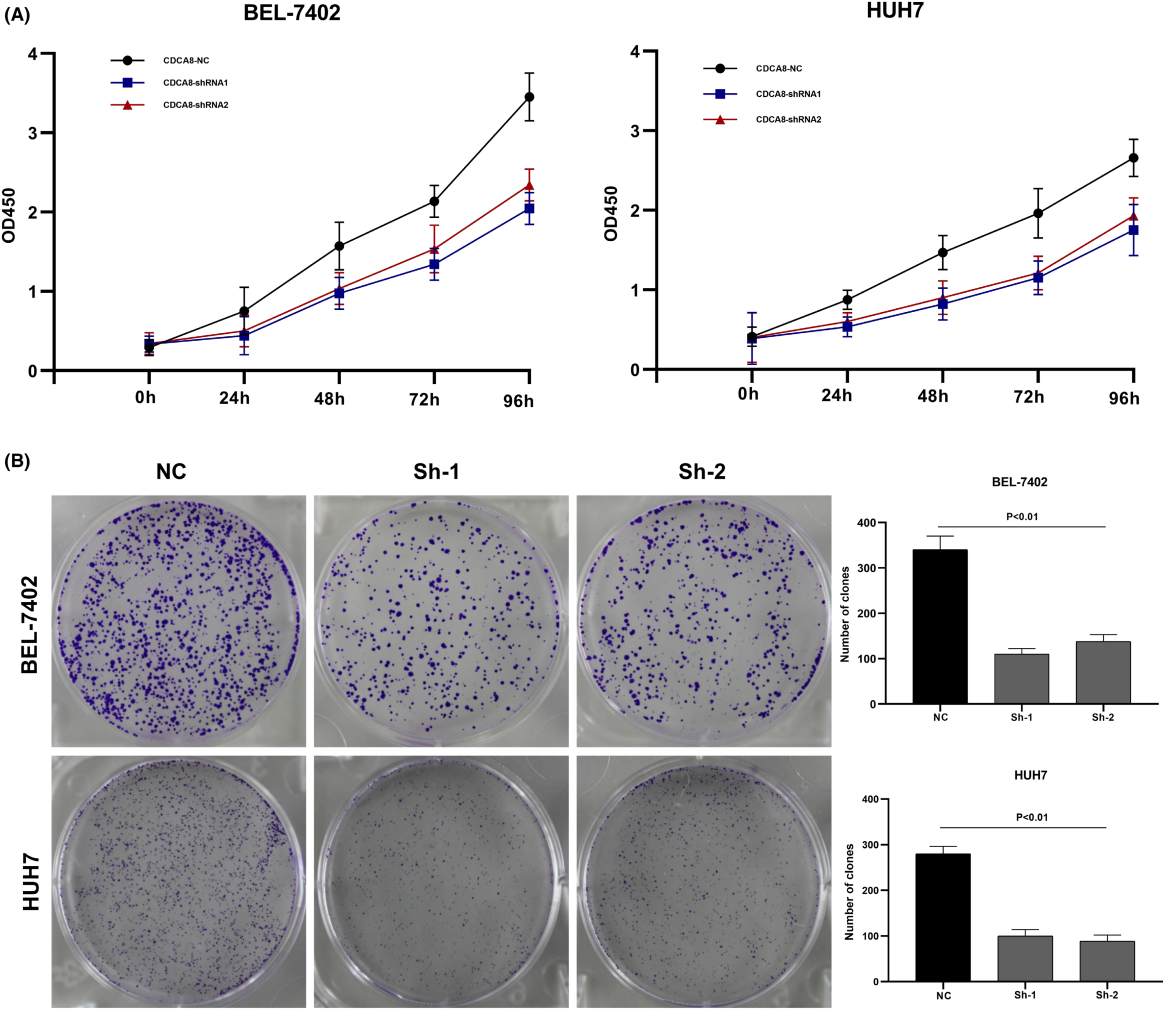
Depletion of Cell division cycle associated 8 (CDCA8) inhibited liver cancer cells proliferation. (A) CCK8 experiments showed that inhibition of CDCA8 could significantly reduce the proliferation of liver cancer cells. (B) cloning experiments showed that inhibition of CDCA8 could significantly reduce the proliferation of liver cancer cells.

**FIGURE 10:**
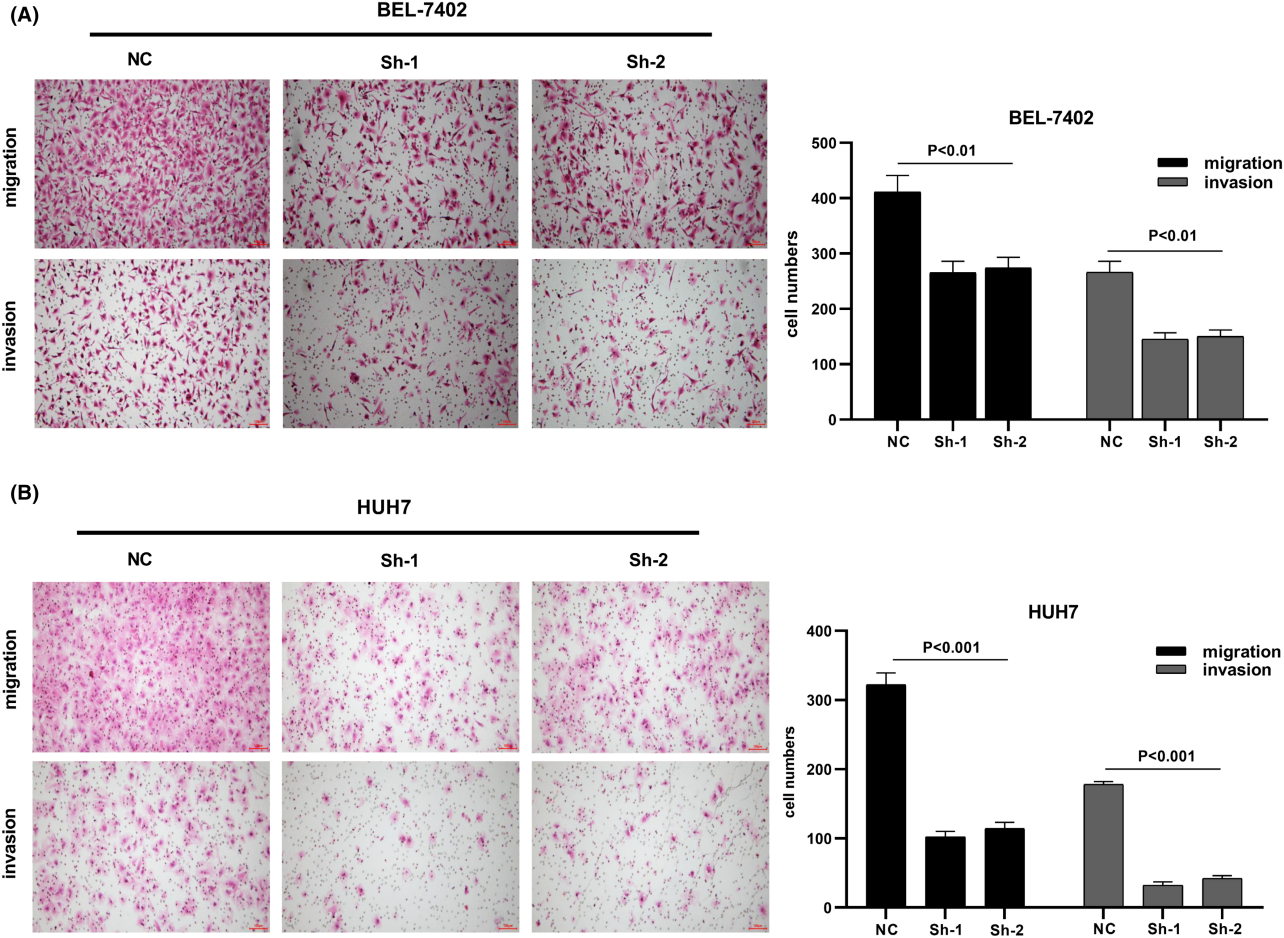
Effect of Cell division cycle associated 8 (CDCA8) knockdown on the migration and invasion of hepatocellular carcinoma (HCC) cells was proved by transwell assays. (A) Migration and invasion of HCC cell line BEL‐7402 were investigated through the use of CDCA8‐sh and CDCA8‐NC. (B) Migration and invasion of HCC cell line HUH‐7 were investigated through the use of CDCA8‐sh and CDCA8‐NC.

**FIGURE 11:**
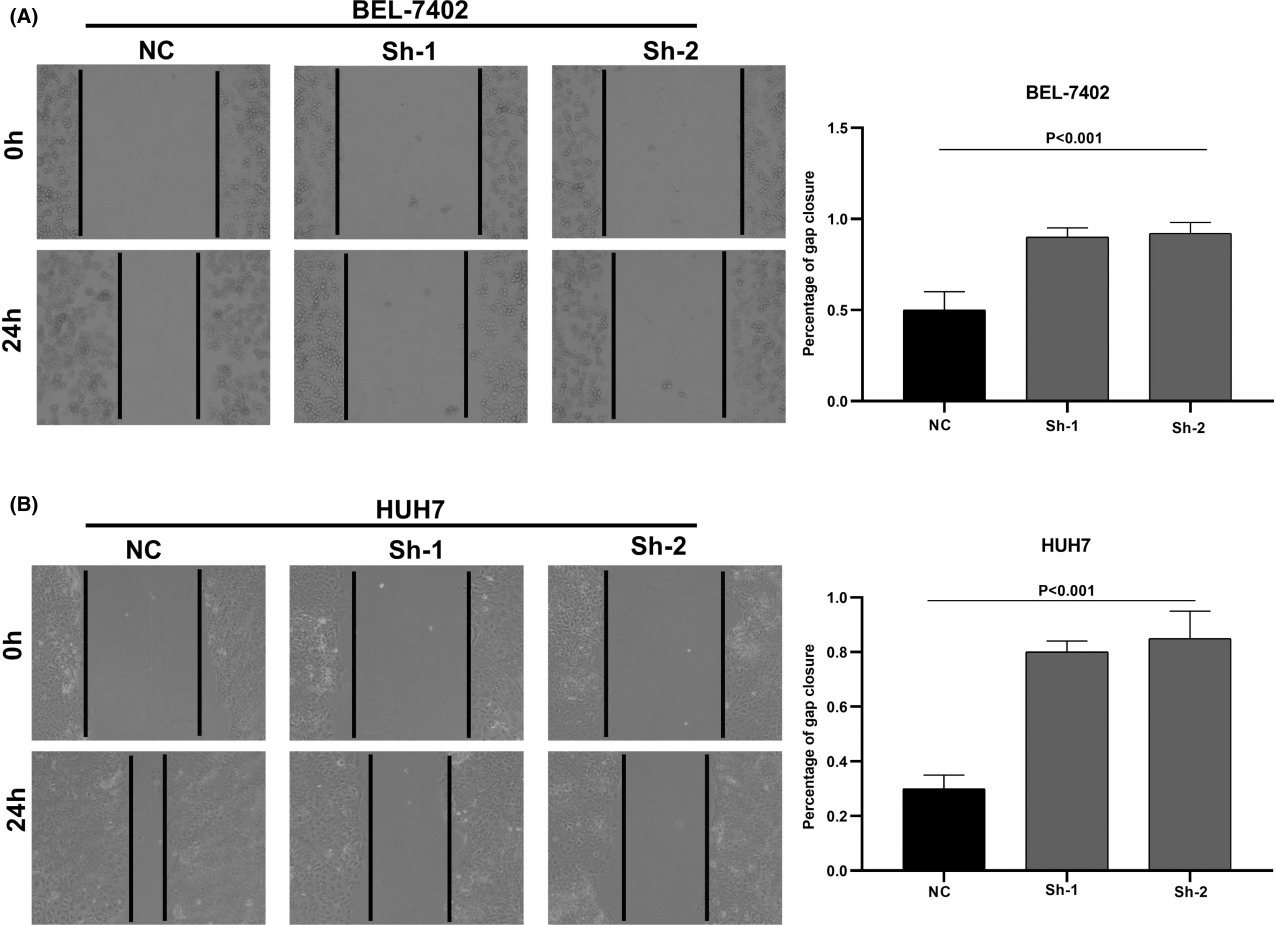
Scratch wound healing assay on hepatocellular carcinoma (HCC) cells. (A) The scratch wound healing assay was used to determine the cell motility ability in HCC cell BEL‐7402 transfected with CDCA8‐sh or CDCA8‐NC. (B) The scratch wound healing assay was used to determine the cell motility ability in HCC cell HUH‐7 transfected with CDCA8‐sh or CDCA8‐NC.

We designed three distinct CDCA8 lentiviral silent infection sequences, and utilized qRT‐PCR to demonstrate the effective silencing of the CDCA8 gene with CDCA8‐sh1 and CDCA8‐sh2 (*p* < 0.001) (Figure S6). CCK8 and cloning experiments were then applied to confirm the CDCA8 significance in liver cancer cell proliferation. We demonstrated that CDCA8 knock down markedly reduced cell proliferation, compared to the NC cells (Figure 9A,B). Next, we conducted transwell assay to assess the migratory and invasive nature of HCC cells harboring the CDCA8 lentivirus and their corresponding NC. HCC cells containing CDCA8 sh1 and sh2 exhibited substantially diminished invasive and migratory capabilities (Figure 10). Moreover, based on our wound healing assay, the motile ability was markedly decreased following CDCA8 silencing (Figure 11).

## 4 CONCLUSION

In conclusion, we assessed CDCA8 expressions in pan‐cancer, and compared its profile among HCC and para‐cancerous tissues. Simultaneously, using qRT‐PCR, western‐blot, and IHC, we verified the significantly high CDCA8 expression in HCC tissues and cells. Cell functional assays revealed that CDCA8 silencing suppressed liver cancer cell proliferation, invasion, and migration. GO term, GSEA, and ssGSEA were performed to identify signaling networks associated with the CDCA8 DEGs, and quantify the extent of CDCA8‐related immune cell invasion. Based on our findings, CDCA8 contributed to the cell cycle, meiosis, NOTCH, PPAR, P53, MYC‐ACTIVE, E2F, and ATM axes. The CDCA8 expression was directly associated with Th2 and T helper cells, and indirectly associated with CD8 T cells, neutrophils, DC, and cytotoxic cells. Additionally, using logistic regression, we demonstrated that CDCA8 not only exhibited marked differential regulation in liver cancer and adjacent tissues, but also showed differences in various subgroups, such as, T stage, PS, TS, HG, and AFP. KM analysis and Cox regression were conducted to assess the link between CDCA8 levels and OS rates. We revealed that elevated CDCA8 expression was a stand‐alone prognostic indicator of worse patient outcome. The ROC revealed that the AUC was 0.997, meaning that CDCA8 can be used as a diagnostic marker for HCC. Lastly, a Cox multivariate analysis‐based nomogram was generated to estimate the influence of CDCA8 on HCC patient prognosis.

## 5 DISCUSSION

CDCA8 is a strong modulator of mitosis and cell division, and it is intricately linked to cancer development and progression.^28^ It is also highly expressed in numerous cancers, and its association with poor patient prognosis indicates its participation in tumorigenesis. A study revealed elevated CDCA8 levels were strongly associated with advanced WHO grade and worse OS and DFS. CDCA8, in combination with E2F1, accelerates glioma proliferation and migration.^11^ In addition, CDCA8 silencing sensitizes ovarian cancer cells to olaparib and cisplatin via induction of the G2/M phase arrest, acceleration of cell apoptosis, enhancement of DNA damage, and interference with RAD51 accumulation in vitro.^12^ CDCA8 knockdown also suppresses T24 and 5637 cell proliferation, migration, and invasion while accelerating apoptosis of bladder cancer cells. CDCA8 is involved in bladder cancer cell cycle modulation. Moreover, using bioinformatics analysis, one study revealed that elevated CDCA8 levels modulate the cell cycle and P53 axes.^29^, ^30^ Moreover, CDCA8 participates in pancreatic ductal adenocarcinoma and lung adenocarcinoma tumor progressions.^31^, ^32^ Based on information from ONCOMINE and GEO, the CDCA8 levels are relatively high in cutaneous melanoma versus normal tissues. Moreover, the elevated CDCA8 expression is correlated with worse outcome in these patients. Of note, CDCA8 silencing inhibits cutaneous melanoma cell line cell proliferation, migration, and invasion. One report revealed that aurora kinase B (AURKB) phosphorylates CDCA8 in vitro, and a simultaneous co‐transactivation of CDCA8 and AURKB is typically seen in multiple cancers. Additionally, the CDCA8 expression and activity regulation is directly linked to the ROCK axis.^10^ Studies revealed that CDCA8 accelerates breast cancer cell cycle progression by suppressing apoptosis, while enhancing tamoxifen resistance and cell proliferation.^33^ Several prior investigations revealed the CDCA8 significance in HCC, with primary focus on the CDCA8‐mediated effect on cell cycle, and its impact on HCC occurrence and development.^22^, ^23^, ^34^ Herein, we demonstrated that CDCA8 modulates HCC progression via immune cell invasion.

The immune microenvironment (IME) is critical for HCC etiology and patient prognosis.^35^, ^36^ Moreover, the HCC microenvironment is a complicated ecosystem composed of several parenchymal cells as well as immune‐associated cells. The verified success of the immune checkpoint suppressive strategies in solid tumors emphasizes the major significance of TME in tumor progression.^37^ The HCC microenvironment includes a majority of immune cell components, such as, the effector T cells, Tregs, and macrophages.^38^ In multiple cancers, the immune cells invasion of tumors is typically linked to an enhanced patient response to immunotherapy and good outcome.^39^ Hepatitis and immune cell invasion of Hepaticus‐infected cancers suggested that chronic inflammation may initially contribute to cancer promotion.^40^ In a majority of cancers, such as, liver cancer, invading immune cells (IICs) serve multifactorial roles, namely, avoidance of immune destruction, activation of tumor cell invasion and metastasis, and angiogenesis induction.^41^ Herein, we demonstrated that CDCA8 was strongly associated with immune invasion. CDCA8 was directly linked to Th2 and helper T cells, and indirectly linked to CD8 T cells, neutrophils, DC, and cytotoxic cells, suggesting that CDCA8 may be essential for TIME.

Based on our current findings and prior research on CDCA8, there is sufficient evidence that CDCA8 serves an essential function as an oncogene. Thus, it has attracted much attention over the last few years. Research have clarified the underlying CDCA8‐based mechanisms from different perspectives. However, its mechanism involving the cell cycle requires further exploration. It is also significant to clarify how CDCA8 affects HCC progression through immune infiltration and TIME. The HCC‐associated immunotherapy is still in its infancy. HCC biomarkers exploration for immunotherapy warrants additional research. At the present time, there are significant unsolved problems to assessing the biomarker‐based prediction of immunotherapeutic efficacy. These can be the focus of future research, which will provide a foundation for CDCA8 usage as an HCC prognostic biomarker.

## AUTHOR CONTRIBUTIONS

**Haomin Wu:** Conceptualization (supporting); formal analysis (lead); investigation (equal); methodology (supporting); project administration (supporting); software (equal); supervision (supporting); validation (equal); visualization (supporting); writing – original draft (lead); writing – review and editing (supporting). **Shiqi Liu:** Conceptualization (supporting); formal analysis (supporting); investigation (equal); methodology (supporting); project administration (supporting); software (equal); supervision (supporting); validation (equal); visualization (lead); writing – original draft (supporting); writing – review and editing (supporting). **Di Wu:** Data curation (equal); investigation (supporting); supervision (supporting); validation (equal); writing – review and editing (supporting). **Haonan Zhou:** Data curation (equal); investigation (supporting); supervision (supporting); validation (equal); writing – review and editing (supporting). **Guoxin Sui:** Data curation (equal); investigation (supporting); supervision (supporting); validation (equal); writing – review and editing (supporting). **Gang Wu:** Conceptualization (lead); funding acquisition (lead); investigation (lead); methodology (lead); project administration (lead); resources (lead); supervision (lead); validation (equal); writing – review and editing (lead).

## FUNDING INFORMATION

Major Science and Technology Special Project in Liaoning Province (2019JH1/10300007).

## CONFLICT OF INTEREST STATEMENT

The authors declare that the research was conducted in the absence of any commercial or financial relationships that could be construed as a potential conflict of interest.

## Supporting information


Table S1:

Table S2:

Figure S1.

Figure S2.

Figure S3.

Figure S4.

Figure S5.

Figure S6.

## Data Availability

The datasets used and/or analyzed during the current study are available from the corresponding author on reasonable request.
