# Supplementary material for: Cell division cycle‐associated 8 is a prognostic biomarker related to immune invasion in hepatocellular carcinoma
Source: Cancer Med. 2023 Feb 28;12(8):10138–55. doi: 10.1002/cam4.5718 (PMC10166956; doi:10.1002/cam4.5718)
Supplement: Supplementary file 1 — Table S1: [file CAM4-12-10138-s004.docx]

**Supplementary table1**: Univariate and multivariate regression (DSS) of prognosis in patients with HCC.

| Characteristics | Total(N) | Univariate analysis | |  | Multivariate analysis | |
| --- | --- | --- | --- | --- | --- | --- |
|  |  | Hazard ratio (95% CI) | P value |  | Hazard ratio (95% CI) | P value |
| T stage | 362 |  |  |  |  |  |
| T1&T2 | 272 | Reference |  |  |  |  |
| T3&T4 | 90 | 3.639 (2.328-5.688) | **<0.001***** |  | 2.072 (0.850-5.052) | 0.109 |
| N stage | 253 |  |  |  |  |  |
| N0 | 249 | Reference |  |  |  |  |
| N1 | 4 | 3.612 (0.870-14.991) | 0.077 |  | 1.194 (0.123-11.612) | 0.878 |
| M stage | 268 |  |  |  |  |  |
| M0 | 265 | Reference |  |  |  |  |
| M1 | 3 | 5.166 (1.246-21.430) | **0.024*** |  | 11.266 (1.929-65.809) | **0.007**** |
| Pathologic stage | 341 |  |  |  |  |  |
| Stage I&Stage II | 254 | Reference |  |  |  |  |
| Stage III&Stage IV | 87 | 3.803 (2.342-6.176) | **<0.001***** |  |  |  |
| Tumor status | 354 |  |  |  |  |  |
| Tumor free | 202 | Reference |  |  |  |  |
| With tumor | 152 | 775790759.389 (0.000-Inf) | 0.994 |  |  |  |
| Histologic grade | 360 |  |  |  |  |  |
| G1&G2 | 227 | Reference |  |  |  |  |
| G3&G4 | 133 | 1.086 (0.683-1.728) | 0.726 |  |  |  |
| AFP(ng/ml) | 275 |  |  |  |  |  |
| <=400 | 214 | Reference |  |  |  |  |
| >400 | 61 | 0.867 (0.450-1.668) | 0.668 |  |  |  |
| Vascular invasion | 309 |  |  |  |  |  |
| No | 204 | Reference |  |  |  |  |
| Yes | 105 | 1.277 (0.707-2.306) | 0.418 |  |  |  |
| Gender | 365 |  |  |  |  |  |
| Female | 118 | Reference |  |  |  |  |
| Male | 247 | 0.813 (0.516-1.281) | 0.373 |  |  |  |
| Age | 365 |  |  |  |  |  |
| <=60 | 174 | Reference |  |  |  |  |
| >60 | 191 | 0.846 (0.543-1.317) | 0.458 |  |  |  |
| Residual tumor | 337 |  |  |  |  |  |
| R0 | 320 | Reference |  |  |  |  |
| R1&R2 | 17 | 1.678 (0.728-3.870) | 0.224 |  |  |  |
| Child-Pugh grade | 235 |  |  |  |  |  |
| A | 214 | Reference |  |  |  |  |
| B&C | 21 | 2.560 (1.123-5.834) | **0.025*** |  | 4.126 (1.443-11.799) | **0.008**** |
| Adjacent hepatic tissue inflammation | 117 |  |  |  |  |  |
| Mild | 100 | Reference |  |  |  |  |
| Severe | 17 | 1.236 (0.418-3.655) | 0.702 |  |  |  |
| CDCA8 | 365 |  |  |  |  |  |
| Low | 183 | Reference |  |  |  |  |
| High | 182 | 2.469 (1.554-3.924) | **<0.001***** |  | 3.575 (1.447-8.832) | **0.006**** |
