# Supplementary material for: Cell division cycle‐associated 8 is a prognostic biomarker related to immune invasion in hepatocellular carcinoma
Source: Cancer Med. 2023 Feb 28;12(8):10138–55. doi: 10.1002/cam4.5718 (PMC10166956; doi:10.1002/cam4.5718)
Supplement: Supplementary file 2 — Table S2: [file CAM4-12-10138-s001.docx]

**Supplementary table2**: Univariate and multivariate regression (PFI) of prognosis in patients with HCC.

| Characteristics | Total(N) | Univariate analysis | |  | Multivariate analysis | |
| --- | --- | --- | --- | --- | --- | --- |
|  |  | Hazard ratio (95% CI) | P value |  | Hazard ratio (95% CI) | P value |
| T stage | 370 |  |  |  |  |  |
| T1&T2 | 277 | Reference |  |  |  |  |
| T3&T4 | 93 | 2.177 (1.590-2.980) | **<0.001***** |  | 0.785 (0.171-3.616) | 0.756 |
| N stage | 258 |  |  |  |  |  |
| N0 | 254 | Reference |  |  |  |  |
| N1 | 4 | 1.370 (0.338-5.552) | 0.659 |  |  |  |
| M stage | 272 |  |  |  |  |  |
| M0 | 268 | Reference |  |  |  |  |
| M1 | 4 | 3.476 (1.091-11.076) | **0.035*** |  | 1.807 (0.505-6.467) | 0.363 |
| Pathologic stage | 349 |  |  |  |  |  |
| Stage I&Stage II | 259 | Reference |  |  |  |  |
| Stage III&Stage IV | 90 | 2.201 (1.591-3.046) | **<0.001***** |  | 1.890 (0.409-8.743) | 0.415 |
| Tumor status | 354 |  |  |  |  |  |
| Tumor free | 202 | Reference |  |  |  |  |
| With tumor | 152 | 11.342 (7.567-17.000) | **<0.001***** |  | 13.923 (8.205-23.627) | **<0.001***** |
| Histologic grade | 368 |  |  |  |  |  |
| G1&G2 | 233 | Reference |  |  |  |  |
| G3&G4 | 135 | 1.152 (0.853-1.557) | 0.355 |  |  |  |
| AFP(ng/ml) | 279 |  |  |  |  |  |
| <=400 | 215 | Reference |  |  |  |  |
| >400 | 64 | 1.045 (0.698-1.563) | 0.832 |  |  |  |
| Vascular invasion | 317 |  |  |  |  |  |
| No | 208 | Reference |  |  |  |  |
| Yes | 109 | 1.676 (1.196-2.348) | **0.003**** |  | 1.485 (0.977-2.257) | 0.064 |
| Gender | 373 |  |  |  |  |  |
| Female | 121 | Reference |  |  |  |  |
| Male | 252 | 0.982 (0.721-1.338) | 0.909 |  |  |  |
| Age | 373 |  |  |  |  |  |
| <=60 | 177 | Reference |  |  |  |  |
| >60 | 196 | 0.960 (0.718-1.284) | 0.783 |  |  |  |
| Residual tumor | 344 |  |  |  |  |  |
| R0 | 326 | Reference |  |  |  |  |
| R1&R2 | 18 | 1.513 (0.840-2.726) | 0.168 |  |  |  |
| Child-Pugh grade | 240 |  |  |  |  |  |
| A | 218 | Reference |  |  |  |  |
| B&C | 22 | 1.395 (0.765-2.545) | 0.277 |  |  |  |
| Adjacent hepatic tissue inflammation | 118 |  |  |  |  |  |
| Mild | 101 | Reference |  |  |  |  |
| Severe | 17 | 1.371 (0.716-2.627) | 0.342 |  |  |  |
| CDCA8 | 373 |  |  |  |  |  |
| Low | 187 | Reference |  |  |  |  |
| High | 186 | 1.768 (1.319-2.368) | **<0.001***** |  | 1.355 (0.905-2.030) | 0.140 |
