# Supplementary figures and images for: Cell division cycle‐associated 8 is a prognostic biomarker related to immune invasion in hepatocellular carcinoma
Source: Cancer Med. 2023 Feb 28;12(8):10138–55. doi: 10.1002/cam4.5718 (PMC10166956; doi:10.1002/cam4.5718)

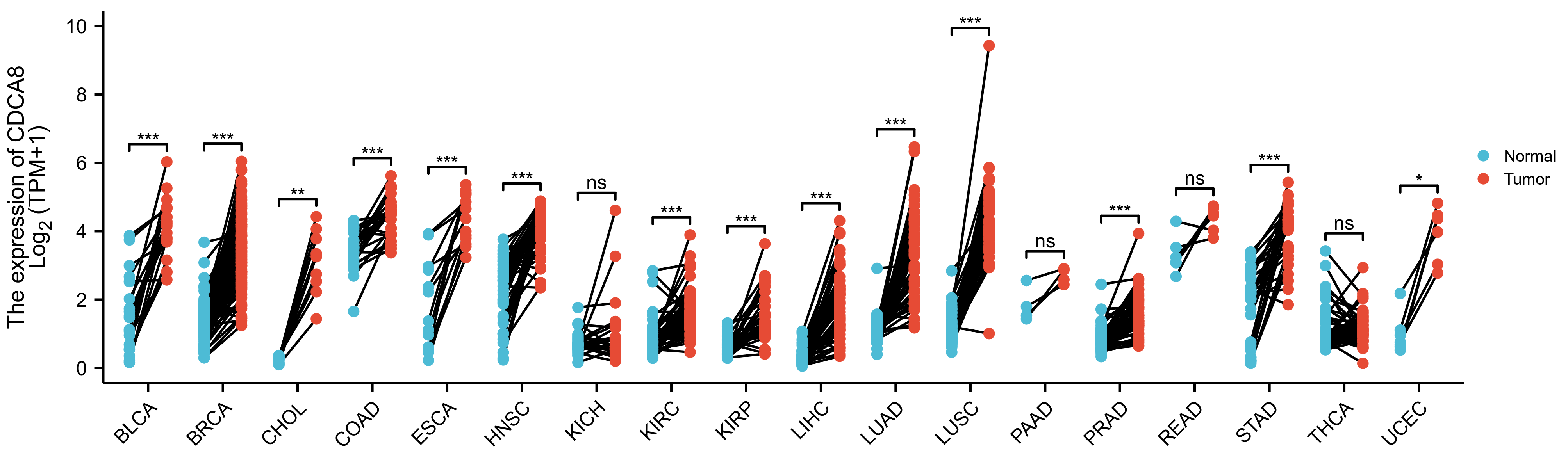

Supplement: Supplementary file 3 — Figure S1. [file CAM4-12-10138-s008.tiff]

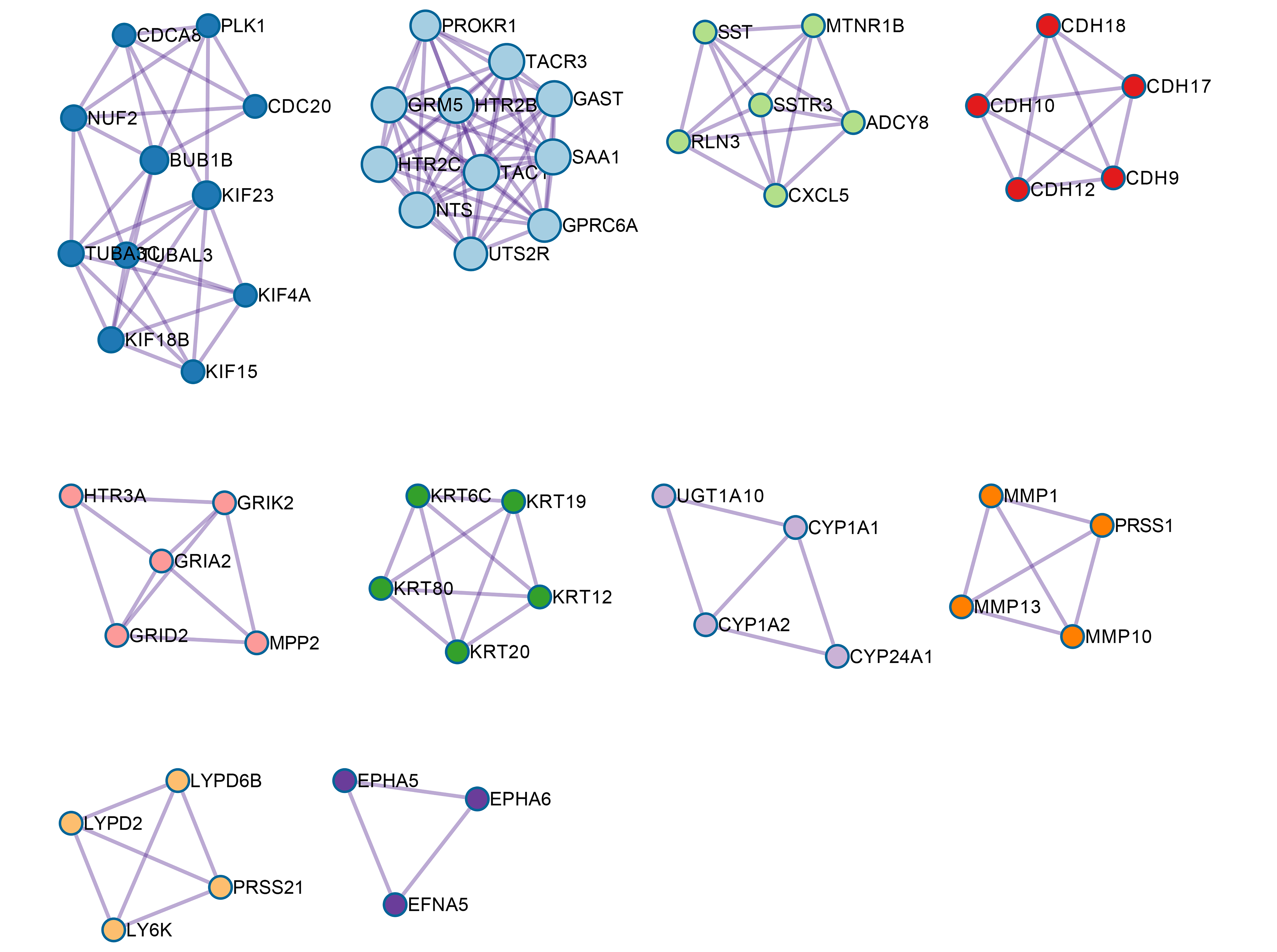

Supplement: Supplementary file 4 — Figure S2. [file CAM4-12-10138-s006.jpg]

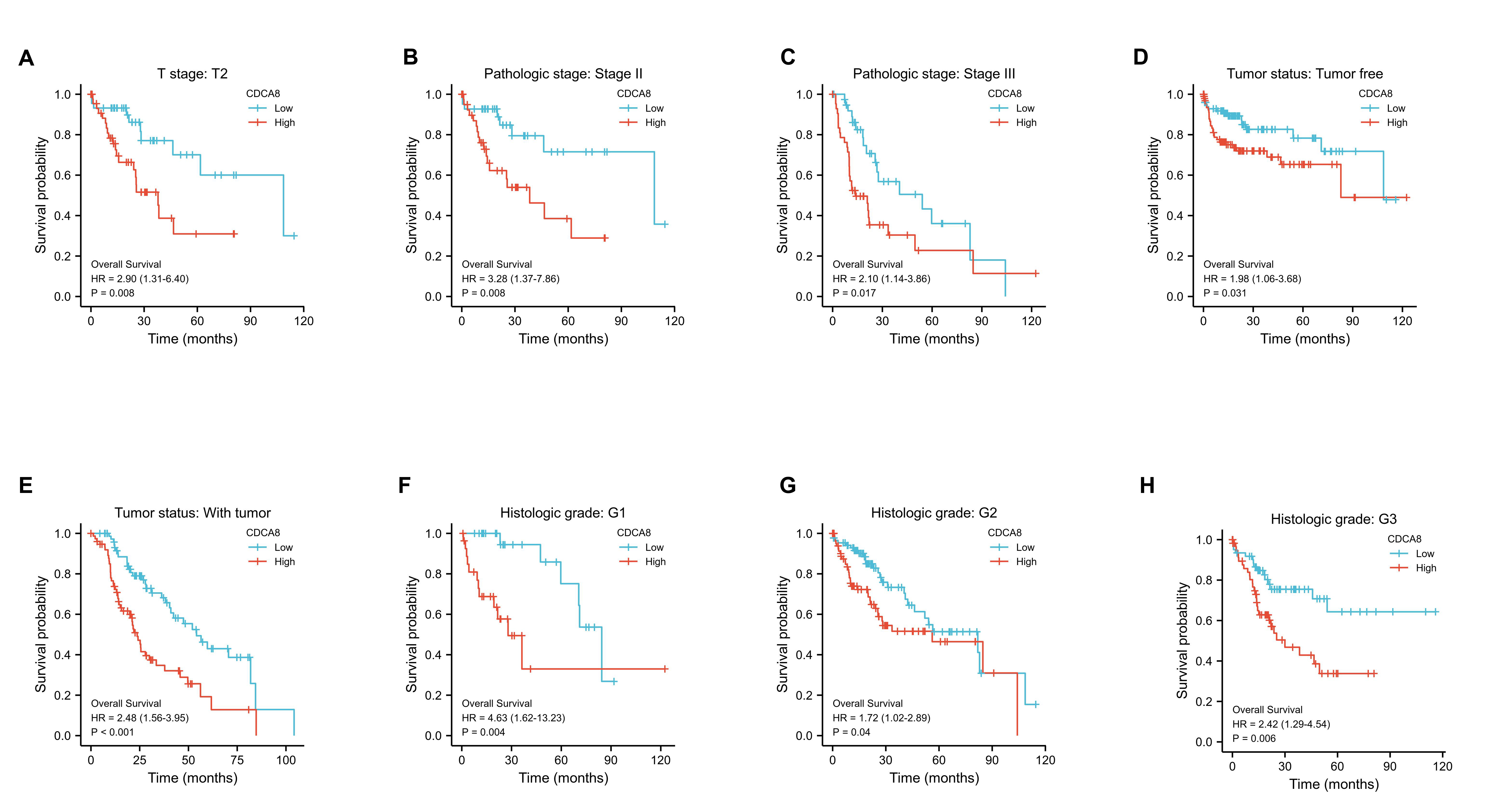

Supplement: Supplementary file 5 — Figure S3. [file CAM4-12-10138-s002.jpg]

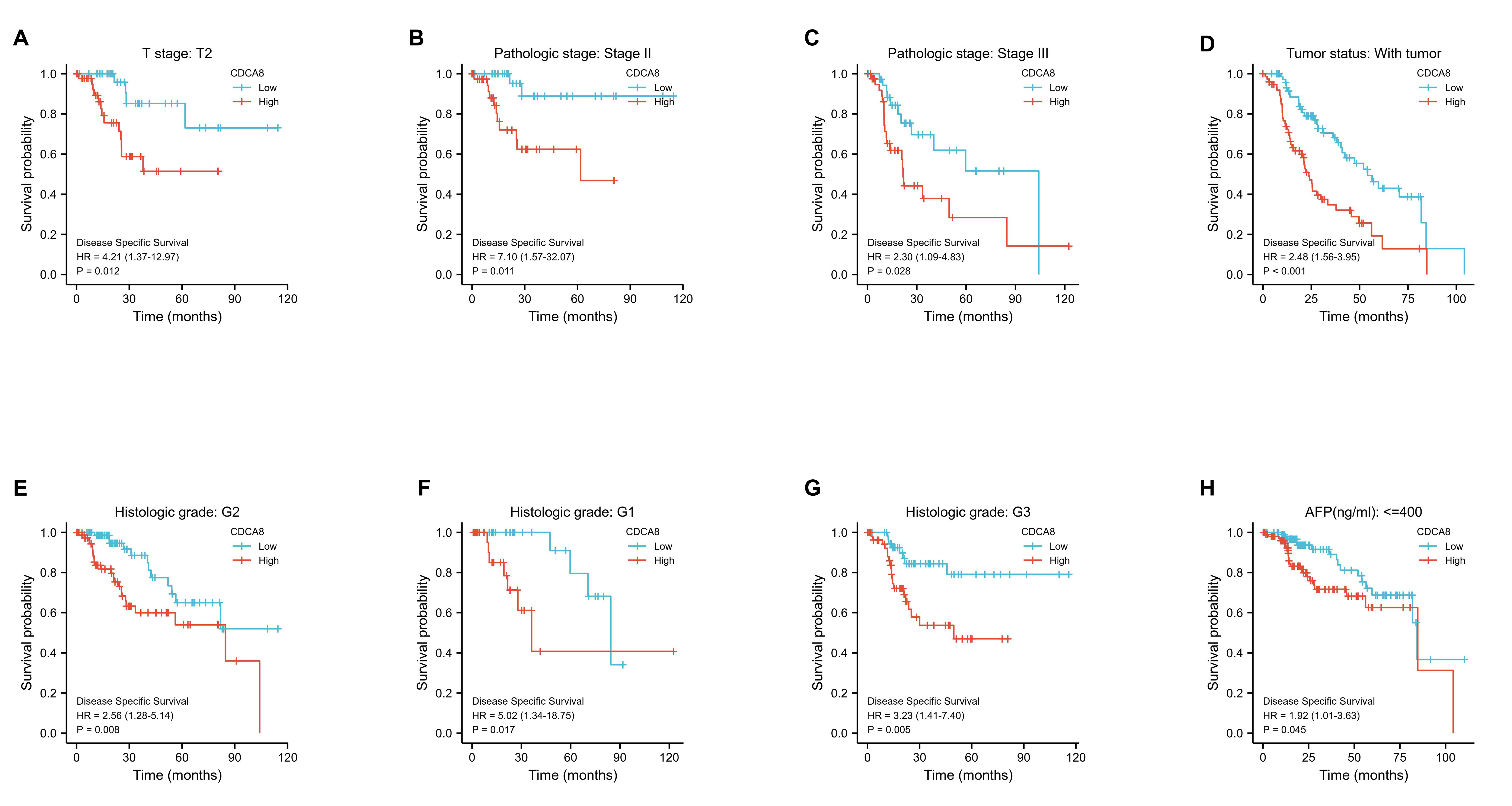

Supplement: Supplementary file 6 — Figure S4. [file CAM4-12-10138-s005.jpg]

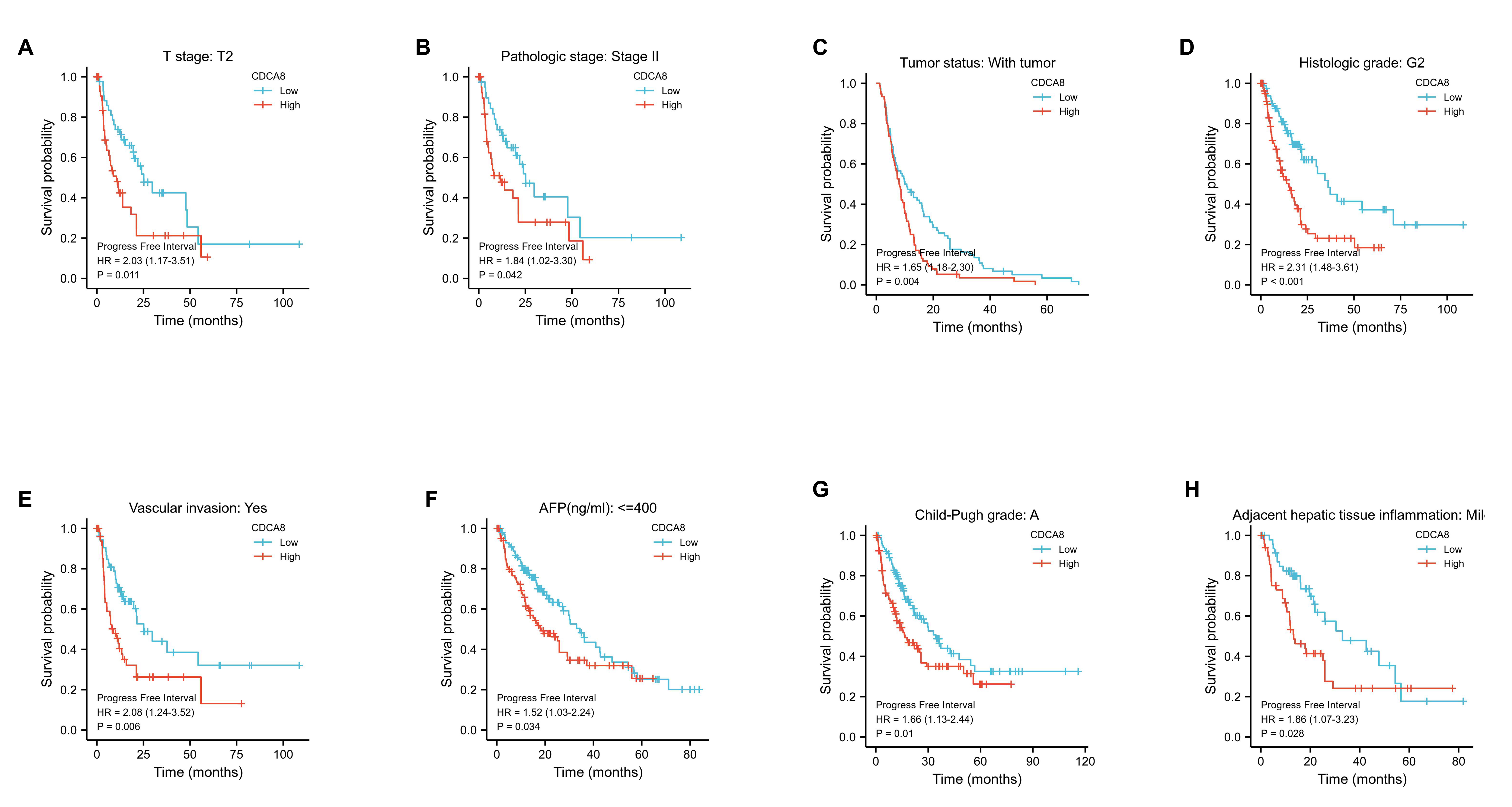

Supplement: Supplementary file 7 — Figure S5. [file CAM4-12-10138-s007.jpg]

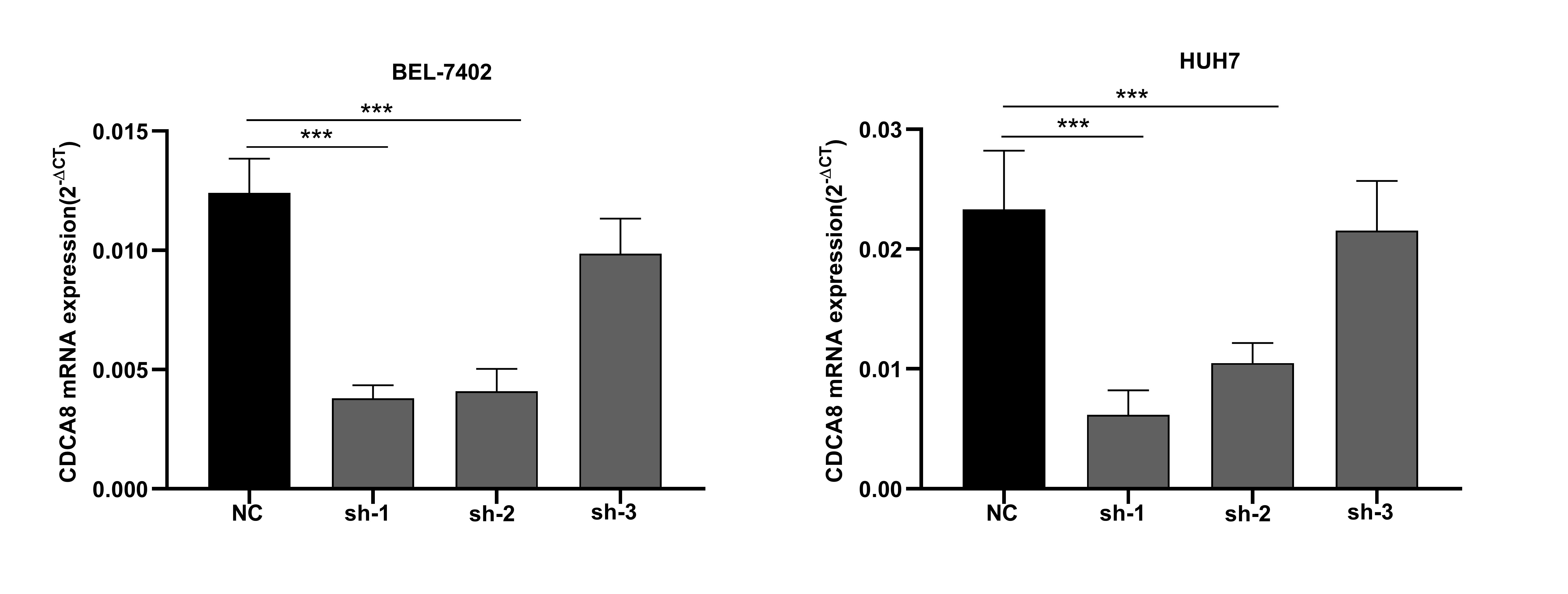

Supplement: Supplementary file 8 — Figure S6. [file CAM4-12-10138-s003.tif]
